# The Effects of Vertebral Body Tethering on the Intervertebral Discs and Facet Joints: A Numerical Analysis

**DOI:** 10.1002/cnm.70084

**Published:** 2025-08-11

**Authors:** Luis Fernando Nicolini, Rafael Carreira Oliveira, Vitor Hugo Tramontini, Marx Ribeiro, Carlos Rodrigo de Mello Roesler, Eduardo Alberto Fancello

**Affiliations:** ^1^ Department of Mechanical Engineering Federal University of Santa Maria Santa Maria Brazil; ^2^ Group of Analysis and Mechanical Design—GRANTE, Department of Mechanical Engineering Federal University of Santa Catarina Florianópolis Brazil; ^3^ Department of Trauma and Reconstructive Surgery University Hospital Halle, Martin‐Luther‐University Halle‐Wittenberg Halle (Saale) Germany; ^4^ Biomechanical Engineering Laboratory—LEBm, Department of Mechanical Engineering Federal University of Santa Catarina Florianópolis Brazil

**Keywords:** curve correction, degeneration, finite element method, scoliosis, spine, vertebral body tethering

## Abstract

Anterior vertebral body tethering (VBT) is a promising technique for the treatment of adolescent idiopathic scoliosis. However, the segments directly treated with VBT can experience substantial loads resulting from the tether pretension, which may alter internal stresses and potentially compromise structures such as the intervertebral discs (IVDs) and facet joints. We aim to investigate the effects of tether within the VBT on the L1–L2 IVD stresses and contact forces of the facet joints, using an extensively calibrated and validated finite element model of the T10–S1 spine. The implant was inserted on the left side of the T10–L3 and tensioned up to 300 N representing the tether pretension applied during surgery and the case of the postoperative neutral position. Subsequently, the spine was tested under an external pure moment of 8 Nm. The tether pretension resulted in a significant increase in the IVD stresses. In the neutral position, a gradual increase in intervertebral pressure (IDP) at the center of the IVD of 0.094, 0.181, and 0.267 MPa was observed after applying forces of 100, 200, and 300 N to the tether, respectively. The contact force of the left facet joint also increased with pretension. It was 12.5 N for the native spine and gradually increased to 49.5, 82.0, and 100.9 N for tether pretensions of 100, 200, and 300 N, respectively, during extension. These results indicate that tether pretension is a key parameter that increases the internal stresses of the IVD and the contact forces of the facet joints at the implant side.

## Graphical abstract

The tether pretension within vertebral body tethering is a key parameter that modifies the stress field of the IVD and the contact forces at the facet joints.

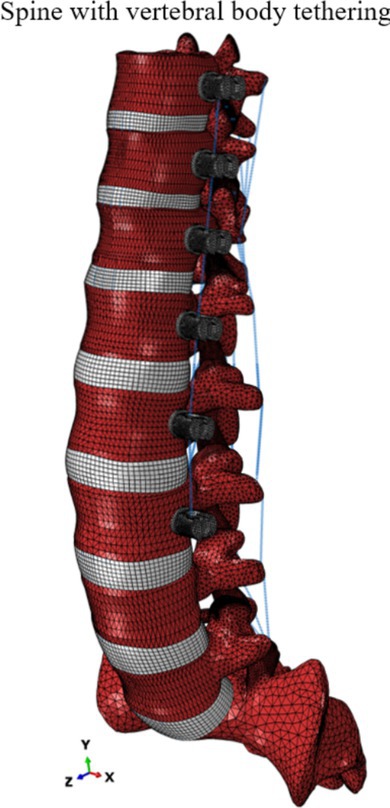

**Abbreviations**
IDP: intradiscal pressure
ROM: range of motion
VBT: vertebral body tethering

## 1 Introduction

Adolescent idiopathic scoliosis (AIS) is a three‐dimensional spinal deformity that occurs at an early age of approximately 11–18 years old [1]. The spine develops a lateral curvature, usually in an elongated “S” or “C” shape in the frontal plane, instead of growing straight [2]. A common conservative treatment for AIS is external bracing, which is recommended by the Scoliosis Research Society for curves between 25° and 40° [3]; it is efficient to alter the natural history of AIS [4, 5]. However, a literature review found low evidence that bracing could be an alternative treatment option for patients above 40° who refused surgery [6]. Moreover, there is still a remarkable percentage of patients who will not benefit from bracing, for many reasons such as pain, skin irritation, and psychosocial issues [6].

Surgery is recommended for patients with severe curves greater than 40°–50° [7]. Posterior fusion is the gold standard for the surgical treatment of AIS, but it has disadvantages such as limiting spinal growth if done before skeletal maturity is achieved, and movement of the fused spinal segments which may contribute to the degeneration of the adjacent segments [8, 9, 10, 11]. A systemic review and meta‐analysis found that nearly half of AIS patients following spinal fusion surgery developed adjacent segment degeneration [12]. The underlying mechanisms of adjacent segment degeneration are not yet fully understood. It has been suggested that, after surgery, patients attempt to regain the same range of motion as before [13], which requires increased motion at the adjacent segments to compensate for the reduced mobility at the fused levels. Adjacent segment degeneration may result from this increased mechanical demand, including abnormal stresses such as increased intradiscal pressure (IDP) on adjacent segments, or it may be a natural aging process not associated with surgery [12, 13].

Anterior vertebral body tethering (VBT) is a novel fusionless technique for the surgical management of skeletally immature patients with AIS [14, 15]. The technique involves the placement of vertebral body screws linked and then tensioned by a flexible cord (tether) to the convexity of the curve to correct the deformity [16]. The system is designed to modulate spinal growth of vertebral bodies according to the Hueter–Volkmann principle, which proposes that growth is retarded by increased mechanical compression and accelerated by reduced loading compared to normal values [17, 18]. With VBT, growth on the tethered convex side of the scoliotic curve is suppressed, while continued growth in the concavity of the curve aims at continued postoperative deformity correction [16]. In addition, VBT surgery is typically performed using an endoscopic technique, which provides patients with a shorter recovery time when compared with posterior spinal fusion [16].

Follow‐up studies in patients have shown that VBT is safe and can correct spinal deformity [19, 20, 21, 22, 23, 24, 25, 26, 27]. Biomechanical cadaveric tests and numerical studies have demonstrated that VBT with a tether pretension of 100 N can preserve some of the spinal mobility in flexion–extension and axial rotation [28, 29, 30]. The same is true for different VBT constructs, such as the double tether and the hybrid technique (one tether and a short rigid rod) considering the global spinal motion [28]. Studies in scoliosis patients indicate that VBT preserves flexion and extension motion at 1 year postoperatively [31, 32]. For Wong et al. [33], VBT resulted in the correction of scoliosis deformity in the coronal and axial planes, with preservation of flexibility. As a motion‐preserving technique, the segments adjacent to the VBT system are theoretically not overloaded and therefore their degeneration is not accelerated by mechanical stresses as it is in fusion. However, the segments directly treated with VBT can experience substantial loads resulting from the tether pretension, with the apical segments being tightened with forces up to 300–400 N [17]. This redistribution may alter internal stresses and potentially compromise structures such as the intervertebral discs (IVDs) and facet joints. Compression of the IVD by tensioning the tether cord poses the theoretical risk of accelerating degenerative changes [34]. This hypothesis was supported by evidence of mild IVD degeneration in IVDs spanned by the tether in a clinical study [34]. In addition, another follow‐up study with nine patients found that one patient developed moderate facet osteoarthritis postoperatively [35]. IVD degeneration has been associated with extreme spinal loading regimens [36] and facet joint degeneration is a known contributor to back pain [37]. Studies suggest that probably any abnormal loading condition, such as overloading, may induce tissue trauma and/or adaptive changes that may lead to IVD degeneration [38]. Therefore, it is important to investigate whether VBT modifies the internal stresses of the IVDs and the facet joints, as this topic needs further verification [39].

We aim to investigate the effects of tether pretension within the VBT on the biomechanics of the spine, including the IDP of the nucleus pulposus, maximum principal stress at various locations of the annulus fibrosus, and contact forces of the facet joints. For that purpose, we used an extensively calibrated and validated finite element model of the spine to understand idiopathic scoliosis with VBT and its effects on the IVD and facet joints.

## 2 Materials and Methods

**FIGURE 1:**
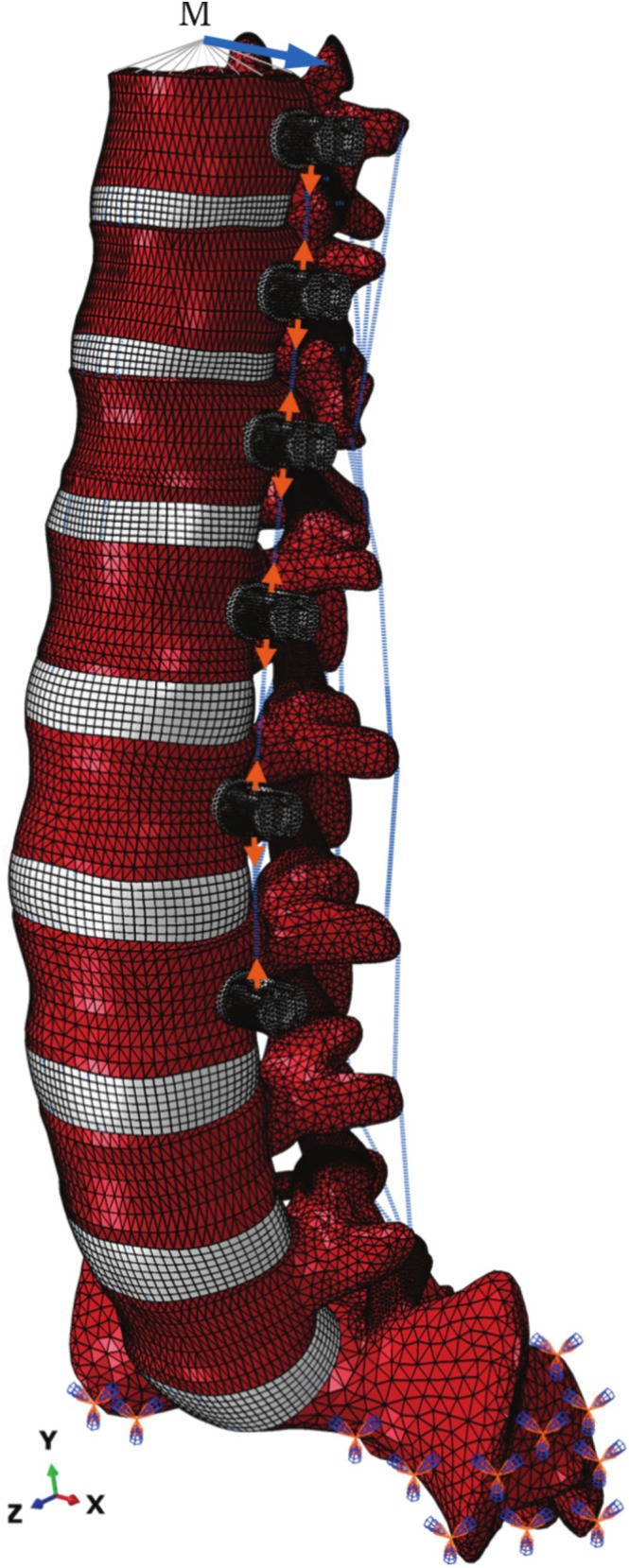
Perspective view of the finite element model of the T10–S1 spine with schematic of the boundary conditions. The sacrum was kept fixed while a pretension is applied to the tether. Subsequently, a pure moment is applied to the T10 vertebra using a point coupled to the vertebra endplate. Flexion–extension, lateral bending, and axial rotation occur about the *x*, *z*, and *y* axis, respectively.

A finite element model of the T10–S1 (Figure 1) was used to perform the simulations and evaluate the effects of VBT with different pretensions.

To represent the nucleus pulposus, a compressible Mooney–Rivlin hyperelastic model was adopted. Its strain energy density function is expressed as:

(1) $$W_{n}=C_{10n}\left(\bar{I}-3\right)+C_{01n}\left(\bar{\mathrm{II}}-3\right)+\frac{1}{D_{n}}{\left(J-1\right)}^{2}$$

where $\bar{I}$ and $\bar{\mathrm{II}}$ denote the first and second invariants of the modified (volume‐preserving) right Cauchy–Green deformation tensor while J represents the volume ratio. The coefficients $C_{10n}$, $C_{01n}$ and $D_{n}$ are the material constants defining matrix stiffness and compressibility [40].

For the annulus fibrosus, we employed the Holzapfel–Gasser–Ogden formulation [41], where the total strain energy function combines an isotropic matrix term with nonlinear fiber contributions.

(2) $$W=C_{10}\left(\bar{I}-3\right)+\frac{1}{D}\left(\frac{J^{2}-1}{2}-\ln J\right)+\frac{K_{1}}{2\left(K_{2}\right)}\sum_{\alpha =1}^{N}\left\{\exp\left[K_{2}{\left\langle E_{\alpha }\right\rangle }^{2}\right]-1\right\}$$

where $C_{10}$ and D characterize the stiffness and compressibility of the annulus ground substance [41]. The fibers' nonlinear stress–strain relationship is parameterized by $K_{1}$ and $K_{2}$, while κ sets the fiber dispersion, and ${\bar{\mathrm{IV}}}_{\left(\alpha \alpha \right)}$ is an invariant equal to the square of the stretch in fiber direction [42].

The model of L1–L2 was calibrated, verified, and validated against experimental cadaveric data [30]. The calibration was performed individually for each spinal structure using experimental data from resection flexibility studies and powerful optimization algorithms which reduced the error between the numerical and experimental results [30]. The model replicated flexion–extension, lateral bending, and axial rotation motions, achieving an average *R* ^2^ value of 0.85 across all loading directions and resection stages. Under combined loading, the model provided an *R* ^2^ ≥ 0.90. Tensile tests of single lamellae from different regions of the annulus fibrosus yielded an *R* ^2^ value of 0.95, closely aligning with experimental results. The model predicted fiber angles of 30° at the anterior aspect of the IVD and 42° at the posterior aspect, closely matching experimental values of 28° and 45°, respectively [43]. The material properties of the IVDs are within the Supporting Information.

The model predicted forces at the facet joints of 0 N for flexion, 5 N for extension, and 4 N for lateral bending, which were within the standard deviation of the experimental values of 2 ± 5, 13 ± 14, and 11 ± 11 N for the respective movements [44]. For axial rotation, the numerical force at the facet joint (79 N) did not match the experimental result (56 ± 17 N) of Niosi et al. [44] but agreed with the result of one specimen (80 N) tested by Wilson et al. [45]. Differences between the numerical and experimental values were expected since they used a different spinal segment (L3–L4) than in our study (L1–L2) and the geometry significantly affects the biomechanics of the spine. Unfortunately, to the best of our knowledge, no experimental study measured the contact forces of the L1–L2 facet joints, which could allow verification of the computational model.

The L1–L2 segment was selected for the analysis of VBT effects because it has been extensively calibrated and validated against in vitro biomechanical data, particularly, for isolated structures such as the IVD, ensuring high simulation accuracy [30, 46, 47]. Moreover, AIS curves frequently apex at L1–L2, highlighting its clinical relevance [28, 48, 49].

The material properties of the L1–L2 segment were extrapolated to other spinal segments, which were then calibrated by adjusting the material parameters of the soft tissue until reaching a good agreement with experimental data from the literature [30, 50, 51, 52, 53, 54, 55, 56, 57, 58, 59] (Supporting Information). The VBT system (Globus Medical Inc., Pennsylvania, USA) was inserted into the left side of the spine from T10 to L3 (Figure 1). The tether of 4 mm diameter, made of polyethylene‐terephthalate (PET), was modeled using a bar element with a Young's modulus of 1500 MPa in tension and negligible resistance to compression [30, 60].

In the first part of this study, the IVD IDP values of the L1–L2 computational model were compared with the experimental data [61] to validate the model. To reproduce the in vitro tests [61] the posterior elements of the vertebrae were removed, the lower endplate of the L2 vertebra was kept fixed, and the midplane of the L1–L2 disc was adjusted to the horizontal. The L1 vertebra was allowed to move only in the vertical direction while loaded in pure axial compression up to 2000 N.

In the second part of this study, simulations were performed to evaluate the effects of VBT following a method as described previously [30, 60]. First, the tether was tensioned, representing the tether pretension applied during surgery and the case of the postoperative neutral position. Operationally, this was performed by translating the force displacement curve representing the material properties of the tether in the displacement axis until it could provide a desired pretension for testing. After applying tether pretensions of 100, 200, or 300 N, the sacrum was kept fixed, and the spine was tested under an external pure moment of 8 Nm to simulate movements of flexion–extension, lateral bending, and axial rotation (Figure 1). In contrast to our previous studies [30, 60] we incorporated multiple spinal segments to better replicate a VBT condition and explored its impact on new variables (IDP and forces at the facet joints).

**FIGURE 2:**
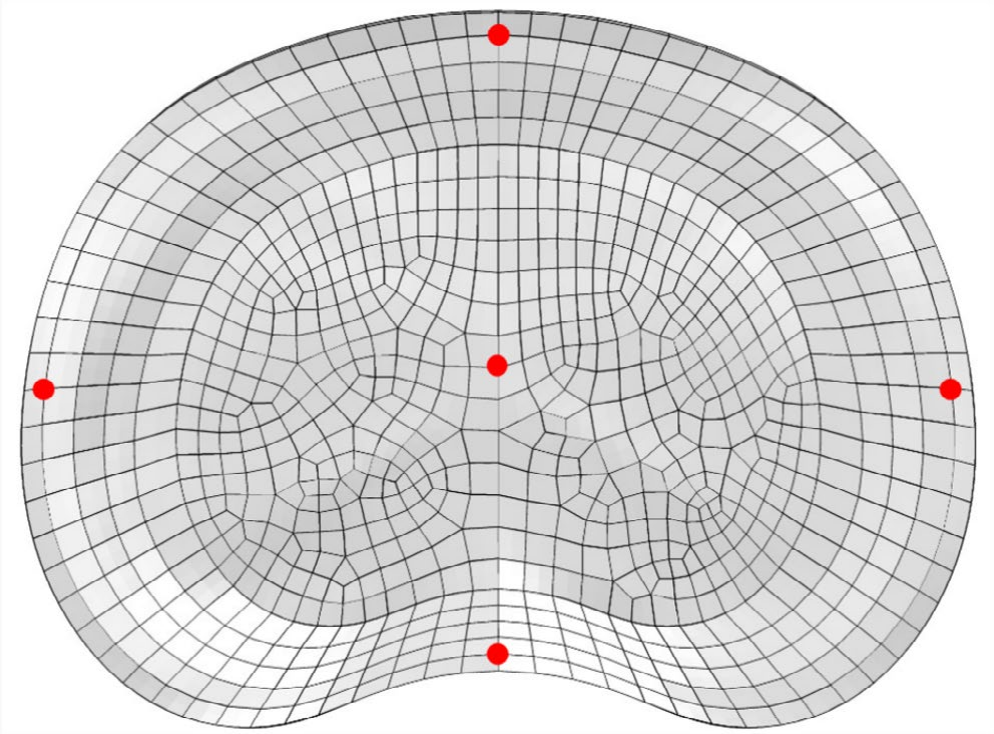
Transverse view of the middle cross‐sectional area of the intervertebral disc showing the five locations (center, left lateral side, right lateral side, ventral side, and dorsal side) considered for analysis. The nucleus pressure and the maximum principal stresses of the annulus fibrosus were evaluated.

The nucleus is a gel‐like substance which the internal pressure exerted by the nucleus pushes the inner margin of the annulus outward during compression [62], caused by body weight and muscle loads. For this reason, the IDP was analyzed at the center of the IVD, as it is widely considered in biomechanical studies [61]. The annulus fibrosus is composed of concentric lamellae of oriented collagen fibers embedded in a hydrated proteoglycan matrix [63] and provides the primary resistance to tensile stress [64]. Therefore, in the present study, we analyzed the maximum principal stress in different locations of the annulus fibrosus (Figure 2).

## 3 Results

### 3.1 Validation of Computational Model

The numerical values of the IDP were 0.31, 0.91, and 1.85 MPa when a pure compressive axial load of 300, 1000, and 2000 N, respectively, was applied. These results agree with experimental data in which the median IDP of 15 lumbar spines were 0.33, 0.95, and 1.85 MPa for the respective load cases [61], corresponding to relative errors of 6.1%, 4.2%, and 0%. Therefore, the model is considered valid for the cases mentioned.

### 3.2 Nucleus Pulposus

**FIGURE 3:**
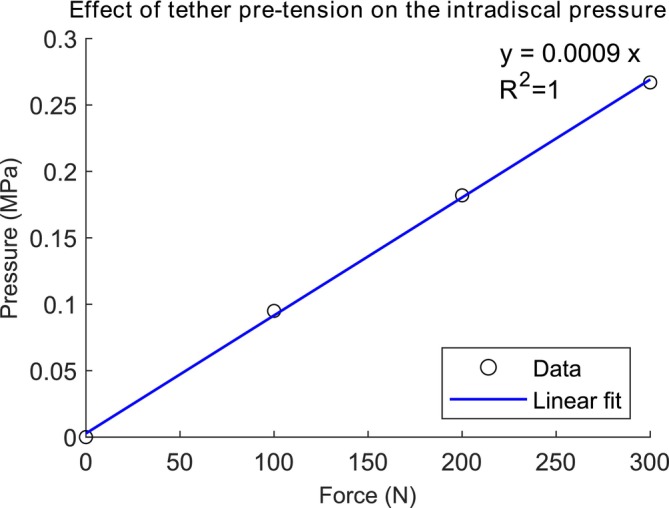
Pressure (MPa) at the center of the L1–L2 intervertebral disc caused by the increase of tether tension within the vertebral body tethering in the neutral position.

In the neutral position, a gradual increase in IDP of 0.094, 0.182, and 0.268 MPa was observed at the center of the IVD after applying forces of 100, 200, and 300 N directly to the tether, respectively (Figure 3). It represents a linear behavior (*R* ^2^ = 1) where the IDP increases at a rate of 0.0009 MPa (0.9 kPa) per unit of force applied to the tether in the neutral position.

**FIGURE 4:**
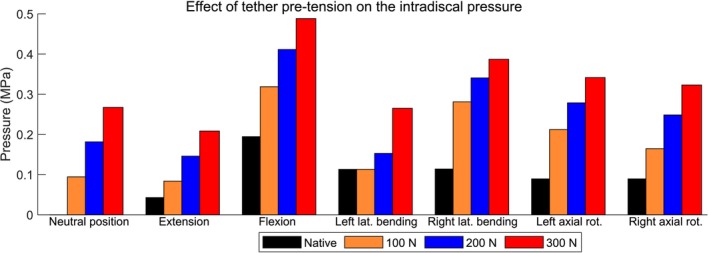
Pressure (MPa) at the center of the intervertebral disc after testing the L1–L2 segment instrumented with different tether pretensions within the vertebral body tethering. The segment was loaded with a pure moment of 8 Nm; except for the neutral position, where no external load was applied.

The increase in tether pretension resulted in an increase in IDP at the center of the IVD not only in the neutral position but also for flexion–extension, lateral bending, and axial rotation (Figure 4 and Table 1). For instance, for extension, the IDP at the center of the L1–L2 IVD was 0.0428 MPa in the native state and gradually increased to 0.0837, 0.146, and 0.209 MPa for initial tether pretensions of 100, 200, and 300 N within the VBT, respectively. The only exception for the IDP increase pattern was the instrumented spine with 100 N in left lateral bending when compared to the native spine. This occurs because the external moment of 8 Nm bent the spine toward the implant side, reducing to zero the tether force and thus its effects on the IDP.

**TABLE 1:** Pressure (MPa) at the center of the intervertebral disc after testing the L1–L2 segment instrumented with different tether pretensions within the vertebral body tethering.

Movement	Pressure for different conditions (MPa)			
	Native spine	100 N	200 N	300 N
Neutral position	0	0.0944	0.1815	0.2674
Extension	0.0428	0.0837	0.1459	0.2085
Flexion	0.1945	0.3187	0.4114	0.4884
Left lateral bending	0.1131	0.1131	0.1528	0.2650
Right lateral bending	0.1139	0.2811	0.3408	0.3870
Left axial rotation	0.0894	0.2121	0.2786	0.3415
Right axial rotation	0.0893	0.1645	0.2485	0.3231

**FIGURE 5:**
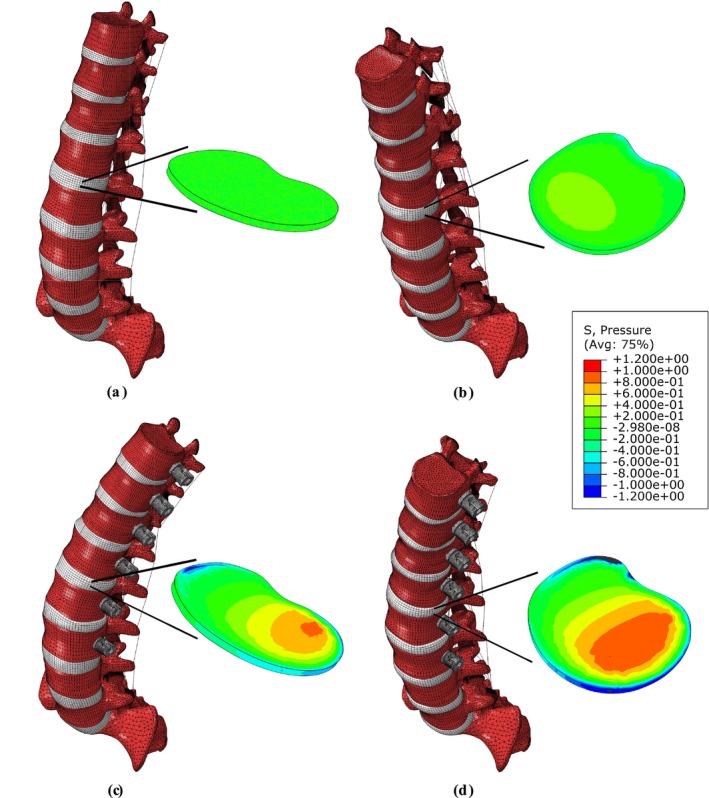
Native spine in the neutral position (a) and under flexion (b). The stress field of the central cross‐sectional area of the L1–L2 intervertebral disc is modified after insertion of a tether pretension of 300 N within vertebral body tethering changes in the neutral (c) and flexion position (d). The tether is represented by a thin line (bar element) connecting the screw heads but was modeled with a diameter of 4 mm.

Figure 5 shows an example of the effects of VBT with a tether pretension of 300 N. In the neutral position, the native spine remained undeformed as no external load was considered (Figure 5a). Under a pure moment of 8 Nm in flexion, the native spine bends forward where the moment induces positive pressure at the mid‐anterior aspect of the IVD (Figure 5). In the neutral position, when a force of 300 N is applied to the tether, the spine bends to the left side (implant side) creating an IVD bulge and significantly altering the pressure field of the IVD (Figure 5c). This pressure field modifies as the spine undergoes flexion (Figure 5d).

### 3.3 Annulus Fibrosus

**FIGURE 6:**
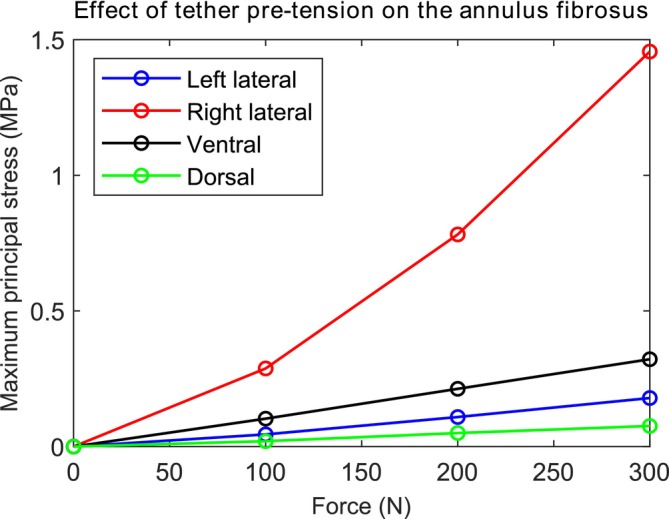
Maximum principal stress (MPa) at the left lateral, right lateral, ventral, and dorsal sides of the L1–L2 intervertebral disc after testing the spine with different tether pretensions within the vertebral body tethering in the neutral position.

The maximum principal stresses at different locations within the annulus fibrosus increased with increasing force applied directly to the tether in the neutral position (Figure 6). For all positions analyzed, the maximum value was 1.456 MPa and occurred on the right lateral side (opposite to the tether). The maximum principal stress values for the other spine motions are shown in Table 2.

**TABLE 2:** Maximum principal stress (MPa) at the left lateral, right lateral, ventral, and dorsal sides of the L1–L2 intervertebral disc after testing the spine with different tether pretensions within the vertebral body tethering.

Movement	Position within disc	Maximum principal stress for different conditions (MPa)			
		Native spine	100 N	200 N	300 N
Neutral position	Left lateral	0.000	0.045	0.109	0.179
	Right lateral	0.000	0.288	0.782	1.456
	Ventral	0.000	0.103	0.213	0.322
	Dorsal	0.000	0.020	0.050	0.076
Extension	Left lateral	0.124	0.177	0.274	0.383
	Right lateral	0.119	0.209	0.565	1.140
	Ventral	0.218	0.203	0.231	0.295
	Dorsal	0.024	0.032	0.043	0.067
Flexion	Left lateral	0.050	0.058	0.080	0.093
	Right lateral	0.047	0.216	0.576	1.106
	Ventral	0.037	0.160	0.284	0.401
	Dorsal	0.551	0.345	0.328	0.374
Left lateral bending	Left lateral	0.047	0.047	0.045	0.071
	Right lateral	1.621	1.621	1.986	2.942
	Ventral	0.485	0.485	0.507	0.591
	Dorsal	0.167	0.167	0.169	0.188
Right lateral bending	Left lateral	1.536	0.248	0.334	0.427
	Right lateral	0.027	0.195	0.389	0.677
	Ventral	0.508	0.328	0.297	0.303
	Dorsal	0.167	0.030	0.042	0.061
Right axial rotation	Left lateral	0.990	0.295	0.195	0.160
	Right lateral	0.443	0.822	1.212	1.639
	Ventral	1.016	1.468	1.401	1.327
	Dorsal	0.054	0.014	0.039	0.064
Left axial rotation	Left lateral	0.451	0.447	0.520	0.611
	Right lateral	1.037	1.649	2.462	3.122
	Ventral	0.985	0.710	0.507	0.439
	Dorsal	0.080	0.037	0.061	0.086

### 3.4 Facet Joints

**FIGURE 7:**
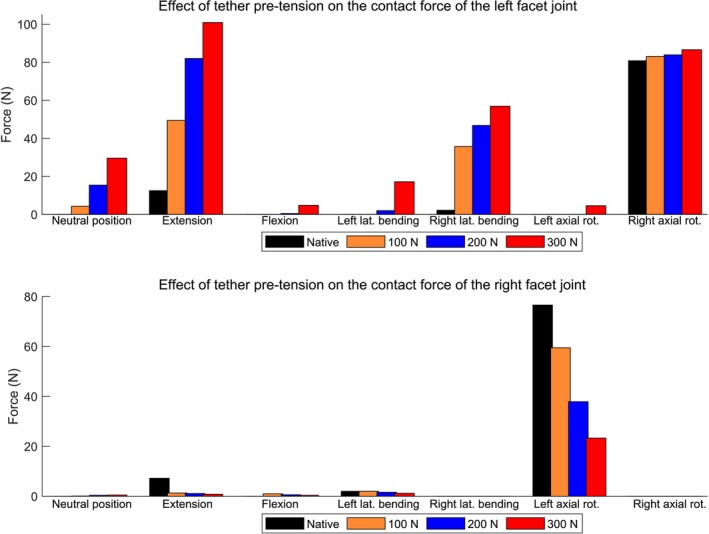
Contact force at the L1–L2 facet joints after testing the spine instrumented with different tether pretensions within the vertebral body tethering. The spine was loaded with a pure moment of 8 Nm except for the neutral position where no external load was applied.

The contact forces acting on the facet joints for different levels of tether pretension and motion directions are shown in Figure 7 and Table 3. For the native spine, the facet joints are unloaded during flexion due to the applied pure moment, which tends to increase the joint gaps. In contrast, during extension, the applied moment approximates the articular surfaces of the facet joints, resulting in contact forces of 12.5 N on the left side and 7.2 N on the right side. The most substantial facet joint loads were observed during axial rotation. In left axial rotation, the right facet joint bears a considerable force (76.6 N), while the left side remains unloaded. Conversely, in right axial rotation, the left facet joint is loaded (80.8 N), with the right side unloaded, indicating a contralateral facet engagement. The insertion of the VBT system alters the facet joint contact forces, as further discussed.

**TABLE 3:** Contact forces of the L1–L2 facet joints after testing the native and instrumented spine with different tether pretension within the vertebral body tethering.

Movement	Position of the facet joint	Force of facet joints (N)			
		Native spine	100 N	200 N	300 N
Neutral position	Left	0.0	4.3	15.4	29.6
	Right	0.0	0.1	0.4	0.5
Extension	Left	12.5	49.5	82.0	100.9
	Right	7.2	1.3	1.1	0.8
Flexion	Left	0.0	0.0	0.5	4.8
	Right	0.0	1.0	0.6	0.4
Left lateral bending	Left	0.0	0.0	2.0	17.2
	Right	2.0	2.0	1.6	1.2
Right lateral bending	Left	2.2	35.7	46.8	56.9
	Right	0.0	0.0	0.0	0.0
Left axial rotation	Left	0.0	0.0	0.0	4.6
	Right	76.6	59.5	37.9	23.3
Right axial rotation	Left	80.8	83.1	83.9	86.6
	Right	0.0	0.0	0.0	0.0

## 4 Discussion

This is the first study to analyze the effects of different tether pretension within VBT on the biomechanics of the L1–L2 spine, including the stresses in the IVD and contact forces of the facet joints. For that purpose, we used an extensively calibrated and validated finite element model of the spine in terms of kinematics, material properties, and contact forces acting on the facet joints. Furthermore, our analysis showed that the average error between the numerical and experimental IDPs at the IVD center was less than 4% (Section 3.1). Therefore, we consider that the computational model provided reliable data for this study.

The required tether pretension within the VBT is defined based on the characteristics of the patient's spine, such as its flexibility and curvature [39]. Our results showed that the tether tension leads to a significant increase in the IDP in the IVD center. For instance, compared to the native spine, an increase in IDP of 0.267 MPa was observed for the neutral position and 0.294 MPa for the extension when instrumented with 300 N of pretension within the VBT (Table 1). For all tested spinal movements, the highest IDP (0.488 MPa) occurred in flexion, which was also the case for the native spine during experimental tests [65, 66]. In addition, it was found that the tether pretension leads to bending of the spine toward the implant side, compresses the IVD, and significantly alters its pressure field (Figure 5). It is expected that the increase in pressure due to the tether will add up to the physiological loads of the patient [39]. Therefore, a significant change in the IDP is expected in patients who underwent VBT surgery, mainly for those who required a relatively large tether pretension within the VBT.

In vivo studies have measured the IDP in the center of the L4–L5 IVD and obtained values of 0.5 MPa for the neutral position, 0.6 MPa for extension, and 1.1 MPa for flexion of the spine [67]. Our study showed that, compared to the native spine, the pretension of 300 N resulted in an IDP increase of 0.267 MPa for the neutral position, 0.166 MPa for extension, and 0.294 MPa for flexion. One may take the in vivo values as a reference and consider that the tether adds pressure to the IVD, assuming the superposition principle. In this case, a tether tension force of 300 N within the VBT would increase the in vivo IDP by 53% for the neutral position, 28% for extension, and 27% for flexion of the spine. However, it is important to acknowledge that the superposition principle is an approximation that assumes linearity in the biomechanical response of the spine. While the linear relationship between tether pretension and IDP in the neutral position of the L1–L2 segment (as observed in Figure 3) may justify its use for pressure estimation within the tested range, this assumption may not be applicable for other parameters, such as the maximum principal stress within the IVD or mechanical responses at different spinal levels—particularly, in the thoracic spine, where the presence of the ribcage introduces nonlinear behavior and additional biomechanical constraints.

To achieve the aforementioned in vivo IDP values, in vitro simulations predicted forces in the muscle erector spine of 170, 100, and 600 N for standing, extension, and flexion of the spine, respectively, while the force in the muscle rectus abdominis offers approximately 20 N [67]. On top of that, the local muscles and the weight of the upper body add a compressive force to the lumbar spine of approximately 200 and 220 N, respectively [67]. Summing up all these loads, the lumbar spine is exposed to compressive forces of approximately 610 N, 540 N, and 1040 N for the cases of standing, extension, and flexion, respectively [68]. Thus, assuming the superposition principle, a tether tension force of 300 N would increase the compressive force acting on the IVD by approximately 49% for the neutral position, 56% for extension, and 29% for flexion. Therefore, from this perspective, the tether can exert a significant compressive force on the IVD.

As an eccentric force, the tether pretension generates not only compression on the IVD but also a moment. It causes lateral bending of the spine and compression of the IVD on the implant side (left side in our study) and traction on the opposite side. Consequently, the maximum principal stresses are relatively large on the right side of the IVD, where the fibers resist traction. On the other hand, the stresses at the dorsal and ventral locations are smaller because they are located close to the neutral line of the moment.

In the native spine, the external moment load causes stretching of the fibers on the ventral side during extension, on the dorsal side during flexion, on the right side during left lateral bending, and at the left side during right lateral bending, leading to an increase in the maximum principal stresses (Table 2). The tether pretension generally accentuates these values. The maximum value was 3.122 MPa and occurred on the right lateral side of the IVD during right axial rotation for the instrumented spine with a tether pretension of 300 N within the VBT. It is significant compared to the maximum principal stress of the native spine (1.037 MPa). For right lateral bending, the instrumentation led to a reduction in the maximum principal stress at the left lateral side of the IVD. This could be explained by the tether resisting most of the stress instead of the left portion of the IVD during right lateral bending.

The insertion of the implant and the application of tether pretension lead to notable modification of the facet joint contact forces, depending on the direction of motion. For the neutral position, the tension applied directly to the tether resulted in left lateral bending of the spine, reducing the gap between the left facet joints and increasing their contact forces. The contact forces at the left facet joint (implant side) increased to 4.3, 15.4, and 29.6 N under pretensions of 100, 200, and 300 N, respectively. In contrast, the right (contralateral) facet joint presented negligible forces (up to 0.5 N). In extension, the tether force substantially modified the contact forces of the left facet joint. It was 12.5 N for the native spine and gradually increased to 49.5, 82.0, and 100.9 N for tether pretensions of 100, 200, and 300 N, respectively (Figure 7a, Table 3). Similarly, during right lateral bending, a substantial increase in contact force at the left facet joint is observed as the pretension escalates, highlighting the asymmetrical nature of loading introduced by the tether system. It was 2.2 N for the native spine and went up to 56.9 N for the maximum tested VBT pretension. A significant contact force was also observed at the left facet joint for the instrumented spine within VBT during right axial rotation. However, it represents an increase of less than 6 N compared to the native situation (Figure 7a, Table 3); that means a variation of 7%. This implies that in right axial rotation, a relatively large force at the left facet joint is naturally occurring in the native spine, and VBT, when inserted on the left side, does not significantly intensify this demand. In contrast, movements such as flexion and left axial rotation result in minor contact forces in the left facet joint, even under maximum pretension. Furthermore, for most of the spinal movements, the increase of the tether pretension tends to decrease the contact force of the right facet joint, since it increases its gap. Overall, the results indicate that while VBT is designed as a motion‐preserving technique, it can introduce considerable posterior joint loading alteration in specific motions, particularly extension and lateral bending, depending on the pretension applied. On the other hand, pretension serves as the primary mechanical driver of spinal remodeling in VBT, where insufficient tension may lead to hypocorrection of spinal curve, while excessive tension can result in overcorrection, depending on the patient‐specific factors.

Facet joint degeneration is a common condition associated with aging and increased joint loading [69]. Studies have shown that facet joint degeneration can be influenced by factors such as facet tropism, asymmetry of the facet joints, and alterations in biomechanics [46, 47]. The degenerative cascade in the lumbar spine typically involves initial degeneration of the IVD followed by facet joint degeneration [70]. Additionally, facet joint degeneration has been linked to changes in articular cartilage, cellular properties of cartilage tissue, and alterations in subchondral bone structure [48, 49]. High stress on the facet joint has been identified as a factor that can induce lumbar facet joint degeneration [71].

The simulated facet joint forces (0–100.9 N) fell within a comparable range (0–171 N) for the L1–L2 presented by other finite element models [72, 73]. This suggests that the simulated facet loads are within physiological limits. However, a limitation of this study is that it remains unknown whether the increase in facet joint forces due to tether pretension could contribute to degeneration.

Jackson et al. [34] IVD health on magnetic resonance imaging (MRI) at 1 year following VBT in AIS patients. Increased degenerative changes in the IVDs spanned by the tether were seen on MRI without evidence of adjacent segment disc disease. Specifically, the mean grade of Pfirrmann, which measures IVD degeneration, was 1.88 preoperatively and increased to 2.31 postoperatively in the IVDs spanned by the tether. This difference was statistically significant (*p* = 0.0075), indicating a measurable change in IVD degeneration. However, the adjacent IVDs that were not directly impacted by the tether showed no significant differences in Pfirrmann grades between preoperative and postoperative imaging. For the IVDs adjacent to the upper instrumented vertebra (UIV), the preoperative Pfirrmann grade was 1.42, which changed to 1.57 postoperatively (*p* = 0.6036). Similarly, for the IVDs adjacent to the lower instrumented vertebra, the preoperative and postoperative Pfirrmann grades remained the same at 2.14 (*p* = 1.000). This suggests that while the IVDs directly spanned by the VBT exhibited increased degeneration post‐surgery, the adjacent IVDs did not show significant changes, indicating a localized impact of the tether on specific IVDs rather than a widespread effect on adjacent areas.

In our study, it was found that the tether pretension results in a significant increase in stresses within the IVD and well changes in the stress field. This may explain the increased degenerative changes in IVDs spanned by the tether found by Jackson et al. [34]. IVD degeneration has been linked in humans to extreme spinal loading regimens [36] and studies suggest that overload can produce tissue trauma and/or adaptive changes that may result in IVD degeneration [38]. Studies have indicated that high gradients of compressive stress within IVD are associated with progressive disc degeneration [55, 56] by altering disc metabolism [74]. This degeneration is characterized by a loss of cellularity, changes in composition, and loss of hydration, leading to changes in disc height and MRI signal density [75]. Furthermore, prolonged abnormal mechanical stress has been shown to accelerate disc cell senescence, impairing the structural and functional homeostasis of IVDs and contributing to IVD degeneration [76].

In contrast, the findings of Yucekul et al. [77] shed light on more comprehensive outcomes following VBT surgery in a larger cohort over a longer follow‐up period of approximately 29 months. Among 21 patients studied in the second postoperative year, a substantial majority (84%) had normal preoperative and follow‐up IVD and facet joint scores across both the operated and adjacent levels. Specifically, 23 patients (92%) had normal intermediate and adjacent IVD grades, suggesting positive outcomes in preserving these segments post‐VBT surgery. While the majority showed stability or improvement, a small percentage showed concerning developments. One patient maintained Grade 4 degeneration in the apical segment, indicating the persistence of severe degeneration. Another patient experienced a change from no preoperative facet degeneration to Grade 2 degeneration in a single adjacent level.

In the study carried out by Yucekul et al. [77] while no new IVD degenerations were noted in most cases, there were cases of deterioration in preexisting degenerated IVDs. One patient had a shift from mild to moderate degeneration, while another patient showcased multilevel degeneration escalating from Grade 2 to Stage 3. The study underscored the need for further investigations in larger cohorts over longer periods of time to elucidate the nuanced effects of relative stabilization and altered biomechanical loads following VBT surgery. Furthermore, Hoernschemeyer et al. [35] findings indicated that, at 2 years post‐VBT, four out of nine patients had a shift of the nucleus pulposus toward the midline in multiple spinal levels, primarily within the tethered region. However, no significant degenerative changes were observed in either the IVDs or posterior facets.

While this numerical study has several limitations mainly regarding the ability of the computational model to represent a scoliotic adolescent spine [30, 46] there is a lack of experimental studies on VBT that could enhance the translational impact. Most of the current literature focuses on clinical outcomes such as curve correction, overcorrection, loss of correction, pulmonary complications, and tether breakage. Additionally, cadaveric biomechanical studies remained limited to changes in the correction of scoliosis and range of motion for different directions and VBT configurations. Therefore, further long‐term experimental and clinical studies focusing on changes in the IVDs and facet joints after VBT with different pretensions are needed to provide clinical recommendations regarding tether pretension to improve the outcome.

## 5 Conclusions

The tether pretension within VBT is a key parameter that modifies the stress field of the IVD. The tether pretension increases the IDP of the nucleus pulposus, the maximum principal stress of the annulus fibrosus, and the contact force of the facet joint at the implant side. Therefore, a significant change in the IVD and facet joints is expected for patients who underwent VBT surgery, mainly for those who required a relatively large tether pretension within VBT.

## Author Contributions

**Luis Fernando Nicolini:** conceptualization, methodology, software, validation, formal analysis, investigation, data curation, writing – original draft preparation, writing – reviewing and editing. **Rafael Carreira Oliveira:** conceptualization, methodology, software, validation, formal analysis, investigation, data curation, writing – original draft preparation, writing – reviewing and editing. **Vitor Hugo Tramontini:** writing – original draft preparation, writing – reviewing and editing. **Marx Ribeiro:** conceptualization, methodology, formal analysis, investigation, data curation, writing – original draft preparation, writing – reviewing and editing. **Carlos Rodrigo de Mello Roesler:** supervision. **Eduardo Alberto Fancello:** supervision. All authors contributed to revising the manuscript and have read and approved the final submitted version.

## Ethics Statement

The authors have nothing to report.

## Conflicts of Interest

The authors declare no conflicts of interest.

## Supporting information


**Data S1:** Supporting Information.

## Data Availability

The data that supports the findings of this study are available in the Supporting Information of this article.

## References

[1] Y. Peng , S.‐R. Wang , G.‐X. Qiu , J.‐G. Zhang , and Q.‐Y. Zhuang , “Research Progress on the Etiology and Pathogenesis of Adolescent Idiopathic Scoliosis,” Chinese Medical Journal 133 (2020): 483–493, 10.1097/CM9.0000000000000652. 31972723 PMC7046244

[2] Z. Šarčević and A. Tepavčević , “Association Between Adolescent Idiopathic Scoliosis and Sacroiliac Joint Dysfunction in Young Athletes,” Medicine 98 (2019): e15161, 10.1097/MD.0000000000015161. 30985695 PMC6485790

[3] S. Negrini , T. M. Hresko , J. P. O'Brien , and N. Price , “Recommendations for Research Studies on Treatment of Idiopathic Scoliosis: Consensus 2014 Between SOSORT and SRS Non–Operative Management Committee,” Scoliosis 10 (2015): 8, 10.1186/s13013-014-0025-4. 25780381 PMC4360938

[4] H. Kuroki , “Brace Treatment for Adolescent Idiopathic Scoliosis,” Journal of Clinical Medicine 7, no. 6 (2018): 136, 10.3390/jcm7060136. 29867010 PMC6024899

[5] Y. Zhang and X. Li , “Treatment of Bracing for Adolescent Idiopathic Scoliosis Patients: A Meta‐Analysis,” European Spine Journal 28 (2019): 2012–2019, 10.1007/s00586-019-06075-1. 31332572

[6] N. Karavidas , “Bracing in the Treatment of Adolescent Idiopathic Scoliosis: Evidence to Date,” Adolescent Health, Medicine and Therapeutics 10 (2019): 153–172, 10.2147/AHMT.S190565. 31632169 PMC6790111

[7] C. Baker , T. Milbrandt , D. Potter , and A. N. Larson , “Anterior Lumbar Vertebral Body Tethering in Adolescent Idiopathic Scoliosis,” Journal of the Pediatric Orthopaedic Society of North America 2 (2020): 12, 10.55275/JPOSNA-2020-145.

[8] G. Mariscal , J. Morales , S. Pérez , et al., “Meta‐Analysis on the Efficacy and Safety of Anterior Vertebral Body Tethering in Adolescent Idiopathic Scoliosis,” European Spine Journal 32 (2023): 140–148, 10.1007/s00586-022-07448-9. 36443510

[9] B. S. Lonner , Y. Ren , V. V. Upasani , et al., “Disc Degeneration in Unfused Caudal Motion Segments Ten Years Following Surgery for Adolescent Idiopathic Scoliosis,” Spine Deformity 6 (2018): 684–690, 10.1016/j.jspd.2018.03.013. 30348344

[10] C. E. Bartley , B. Yaszay , T. P. Bastrom , et al., “Perioperative and Delayed Major Complications Following Surgical Treatment of Adolescent Idiopathic Scoliosis,” Journal of Bone and Joint Surgery 99 (2017): 1206–1212, 10.2106/JBJS.16.01331. 28719560

[11] B. Wilk , L. A. Karol , C. E. Johnston , S. Colby , and N. Haideri , “The Effect of Scoliosis Fusion on Spinal Motion: A Comparison of Fused and Nonfused Patients With Idiopathic Scoliosis,” Spine 31 (2006): 309–314, 10.1097/01.brs.0000197168.11815.ec. 16449904

[12] F. Liu , F. Liu , and H. Wang , “Half of the Adolescent Idiopathic Scoliosis Patients May Have Lumbar Adjacent Segment Degeneration Following Spinal Fusion: A Systemic Review and Meta‐Analysis,” Journal of Orthopaedic Surgery 32 (2024): 10225536241248711, 10.1177/10225536241248711. 38647667

[13] T. Akamaru , N. Kawahara , S. T. Yoon , et al., “Adjacent Segment Motion After a Simulated Lumbar Fusion in Different Sagittal Alignments—A Biomechanical Analysis,” Spine 28 (2003): 1560–1566. 12865845

[14] A. Baroncini and A. Courvoisier , “The Different Applications of Vertebral Body Tethering—Narrative Review and Clinical Experience,” Journal of Orthopaedics 37 (2023): 86–92, 10.1016/j.jor.2023.02.012. 36974090 PMC10039119

[15] S. Martin , N. Cobetto , A. N. Larson , and C.‐E. Aubin , “Biomechanical Modeling and Assessment of Lumbar Vertebral Body Tethering Configurations,” Spine Deformity 11 (2023): 1041–1048, 10.1007/s43390-023-00697-8. 37179281

[16] K. L. Mulford , C. Regan , C. P. Nolte , Z. W. Pinter , T. A. Milbrandt , and A. N. Larson , “Automated Measurements of Interscrew Angles in Vertebral Body Tethering Patients With Deep Learning,” Spine Journal 24 (2023): 333–339, 10.1016/j.spinee.2023.09.011. 37774982

[17] A. Raitio , J. Syvänen , and I. Helenius , “Vertebral Body Tethering: Indications, Surgical Technique, and a Systematic Review of Published Results,” Journal of Clinical Medicine 11 (2022): 2576, 10.3390/jcm11092576. 35566702 PMC9099651

[18] I. A. F. Stokes , “Mechanical Effects on Skeletal Growth,” Journal of Musculoskeletal & Neuronal Interactions 2 (2022): 277–280. 15758453

[19] F. Miyanji , J. Pawelek , L. A. Nasto , A. Simmonds , and S. Parent , “Safety and Efficacy of Anterior Vertebral Body Tethering in the Treatment of Idiopathic Scoliosis,” Bone & Joint Journal 102‐B (2020): 1703–1708, 10.1302/0301-620X.102B12.BJJ-2020-0426.R1. PMC7954148 33249889

[20] M. Boudissa , A. Eid , E. Bourgeois , J. Griffet , and A. Courvoisier , “Early Outcomes of Spinal Growth Tethering for Idiopathic Scoliosis With a Novel Device: A Prospective Study With 2 Years of Follow‐Up,” Child's Nervous System 33 (2017): 813–818, 10.1007/s00381-017-3367-4. 28324184

[21] T. C. McDonald , S. A. Shah , J. B. Hargiss , et al., “When Successful, Anterior Vertebral Body Tethering (VBT) Induces Differential Segmental Growth of Vertebrae: An In Vivo Study of 51 Patients and 764 Vertebrae,” Spine Deformity 10 (2022): 791–797, 10.1007/s43390-022-00471-2. 35064912

[22] J. Bernard , T. Bishop , J. Herzog , et al., “Dual Modality of Vertebral Body Tethering,” Bone & Joint Open 3 (2022): 123–129, 10.1302/2633-1462.32.BJO-2021-0120.R1. 35119295 PMC8886322

[23] T. Pehlivanoglu , I. Oltulu , Y. Erdag , et al., “Comparison of Clinical and Functional Outcomes of Vertebral Body Tethering to Posterior Spinal Fusion in Patients With Adolescent Idiopathic Scoliosis and Evaluation of Quality of Life: Preliminary Results,” Spine Deformity 9 (2021): 1175–1182, 10.1007/s43390-021-00323-5. 33683642

[24] T. D. P. von Treuheim , L. Eaker , J. Markowitz , D. Shankar , J. Meyers , and B. Lonner , “Anterior Vertebral Body Tethering for Scoliosis Patients With and Without Skeletal Growth Remaining: A Retrospective Review With Minimum 2‐Year Follow‐Up,” International Journal of Spine Surgery 17 (2023): 6–16, 10.14444/8357. 36113952 PMC10025839

[25] M. E. Boeyer , S. Farid , S. Wiesemann , and D. G. Hoernschemeyer , “Outcomes of Vertebral Body Tethering in the Lumbar Spine,” Spine Deformity 11 (2023): 909–918, 10.1007/s43390-023-00662-5. 36820998

[26] N. A. Pulido , M. G. Vitale , S. Parent , et al., “Vertebral Body Tethering for Non‐Idiopathic Scoliosis: Initial Results From a Multicenter Retrospective Study,” Spine Deformity 11 (2023): 139–144, 10.1007/s43390-022-00575-9. 36070136

[27] J. W. Siu , H.‐H. Wu , S. Saggi , S. Allahabadi , T. Katyal , and M. Diab , “Radiographic and Perioperative Outcomes Following Anterior Thoracic Vertebral Body Tethering and Posterior Lumbar Spine Tethering: A Pilot Series,” Spine Deformity 11 (2023): 1399–1408, 10.1007/s43390-023-00717-7. 37355490 PMC10587020

[28] L. F. Nicolini , P. Kobbe , J. Seggewiß , et al., “Motion Preservation Surgery for Scoliosis With a Vertebral Body Tethering System: A Biomechanical Study,” European Spine Journal 31 (2022): 1013–1021, 10.1007/s00586-021-07035-4. 34716821

[29] P. Trobisch , J. M. Mahoney , E. K. Eichenlaub , et al., “An Investigation of Range of Motion Preservation in Fusionless Anterior Double Screw and Cord Constructs for Scoliosis Correction,” European Spine Journal 32 (2023): 1173–1186, 10.1007/s00586-023-07608-5. 36871254

[30] L. F. Nicolini , J. Greven , P. Kobbe , et al., “The Effects of Tether Pretension Within Vertebral Body Tethering on the Biomechanics of the Spine: A Finite Element Analysis,” Latin American Journal of Solids and Structures 19 (2022): 1–17, 10.1590/1679-78256932.

[31] S. E. Mathew , T. A. Milbrandt , and A. N. Larson , “Measurable Lumbar Motion Remains 1 Year After Vertebral Body Tethering,” Journal of Pediatric Orthopaedics 42 (2022): e861–e867, 10.1097/BPO.0000000000002202. 35878415

[32] A. F. Buyuk , T. A. Milbrandt , S. E. Mathew , and A. N. Larson , “Measurable Thoracic Motion Remains at 1 Year Following Anterior Vertebral Body Tethering, With Sagittal Motion Greater Than Coronal Motion,” Journal of Bone and Joint Surgery 103 (2021): 2299–2305, 10.2106/JBJS.20.01533. 34270505

[33] H.‐K. Wong , J. N. M. Ruiz , P. O. Newton , and K.‐P. G. Liu , “Non‐Fusion Surgical Correction of Thoracic Idiopathic Scoliosis Using a Novel, Braided Vertebral Body Tethering Device,” JBJS Open Access 4 (2019): e0026, 10.2106/JBJS.OA.19.00026. 32043058 PMC6959921

[34] T. J. Jackson , T. A. Milbrandt , S. E. Mathew , J. A. Heilman , and A. N. Larson , “Intervertebral Disk Health Following Vertebral Body Tethering for Adolescent Idiopathic Scoliosis: A Preliminary Study,” Journal of Pediatric Orthopaedics 42 (2022): 347–353, 10.1097/BPO.0000000000002172. 35543608

[35] D. G. Hoernschemeyer , M. E. Boeyer , N. M. Tweedy , J. R. Worley , and J. R. Crim , “A Preliminary Assessment of Intervertebral Disc Health and Pathoanatomy Changes Observed Two Years Following Anterior Vertebral Body Tethering,” European Spine Journal 30 (2021): 3442–3449, 10.1007/s00586-021-06972-4. 34494139

[36] J. C. Lotz , A. H. Hsieh , A. L. Walsh , E. I. Palmer , and J. R. Chin , “Mechanobiology of the Intervertebral Disc,” Biochemical Society Transactions 30 (2002): 853–858, 10.1042/bst0300853. 12440932

[37] D. G. Bisson , P. Lama , F. Abduljabbar , et al., “Facet Joint Degeneration in Adolescent Idiopathic Scoliosis,” JOR Spine 1 (2018): e1016, 10.1002/jsp2.1016. 31463443 PMC6686828

[38] I. A. F. Stokes and J. C. Iatridis , “Mechanical Conditions That Accelerate Intervertebral Disc Degeneration: Overload Versus Immobilization,” Spine 29 (2004): 2724–2732, 10.1097/01.brs.0000146049.52152.da. 15564921 PMC7173624

[39] V. Lalande , I. Villemure , M. Vonthron , S. Parent , and C.‐É. Aubin , “Cyclically Controlled Vertebral Body Tethering for Scoliosis: An In Vivo Verification in a Pig Model of the Pressure Exerted on Vertebral End Plates,” Spine Deformity 8 (2020): 39–44, 10.1007/s43390-019-00021-3. 31981151

[40] M. Gurtin , An Introduction to Continuum Mechanics, 1st ed. (Academic Press, 1982).

[41] G. A. Holzapfel , T. C. Gasser , and R. W. Ogden , “A New Constitutive Framework for Arterial Wall Mechanics and a Comparative Study of Material Models,” Journal of Elasticity 61 (2000): 1–48, 10.1023/A:1010835316564.

[42] T. C. Gasser , R. W. Ogden , and G. A. Holzapfel , “Hyperelastic Modelling of Arterial Layers With Distributed Collagen Fibre Orientations,” Journal of the Royal Society Interface 3 (2006): 15–35, 10.1098/rsif.2005.0073. 16849214 PMC1618483

[43] J. J. Cassidy , A. Hiltner , and E. Baer , “Hierarchical Structure of the Intervertebral Disc,” Connective Tissue Research 23 (1989): 75–88, 10.3109/03008208909103905. 2632144

[44] C. A. Niosi , D. C. Wilson , Q. Zhu , O. Keynan , D. R. Wilson , and T. R. Oxland , “The Effect of Dynamic Posterior Stabilization on Facet Joint Contact Forces: An In Vitro Investigation,” Spine 33 (2008): 19–26, 10.1097/BRS.0b013e31815e7f76. 18165744

[45] D. C. Wilson , C. a. Niosi , Q. a. Zhu , T. R. Oxland , and D. R. Wilson , “Accuracy and Repeatability of a New Method for Measuring Facet Loads in the Lumbar Spine,” Journal of Biomechanics 39 (2006): 348–353, 10.1016/j.jbiomech.2004.12.011. 16321637

[46] L. F. Nicolini , R. C. Oliveira , M. Ribeiro , et al., “Tether Pre‐Tension Within Vertebral Body Tethering Reduces Motion of the Spine and Influences Coupled Motion: A Finite Element Analysis,” Computers in Biology and Medicine 169 (2024): 107851, 10.1016/j.compbiomed.2023.107851. 38113683

[47] M. Atad , G. Gruber , M. Ribeiro , et al., “Neural Network Surrogate and Projected Gradient Descent for Fast and Reliable Finite Element Model Calibration: A Case Study on an Intervertebral Disc,” Computers in Biology and Medicine 186 (2025): 109646, 10.1016/j.compbiomed.2024.109646. 39787664

[48] K. Chen , X. Zhai , T. Zhou , et al., “Characteristics Analysis of Segmental and Regional Lumbar Spontaneous Compensation Post Thoracic Fusion in Lenke 1 and 2 Adolescent Idiopathic Scoliosis,” BMC Musculoskeletal Disorders 22 (2021): 935, 10.1186/s12891-021-04821-5. 34758789 PMC8582136

[49] C. Slattery and K. Verma , “Classifications in Brief: The Lenke Classification for Adolescent Idiopathic Scoliosis,” Clinical Orthopaedics and Related Research 476 (2018): 2271–2276, 10.1097/CORR.0000000000000405. 30179943 PMC6259994

[50] M. Couvertier , A. Germaneau , M. Saget , et al., “Biomechanical Analysis of the Thoracolumbar Spine Under Physiological Loadings: Experimental Motion Data Corridors for Validation of Finite Element Models,” Proceedings of the Institution of Mechanical Engineers. Part H 231 (2017): 975–981, 10.1177/0954411917719740. 28707505

[51] A. Germaneau , T. Vendeuvre , M. Saget , et al., “A Novel Approach for Biomechanical Spine Analysis: Mechanical Response of Vertebral Bone Augmentation by Kyphoplasty to Stabilise Thoracolumbar Burst Fractures,” Journal of the Mechanical Behavior of Biomedical Materials 59 (2016): 291–303, 10.1016/j.jmbbm.2016.02.002. 26896762

[52] Y. Guan , N. Yoganandan , J. Moore , et al., “Moment‐Rotation Responses of the Human Lumbosacral Sinal Column,” Journal of Biomechanics 40 (2007): 1975–1980, 10.1016/j.jbiomech.2006.09.027. 17101141

[53] F. Heuer , H. Schmidt , Z. Klezl , L. Claes , and H.‐J. Wilke , “Stepwise Reduction of Functional Spinal Structures Increase Range of Motion and Change Lordosis Angle,” Journal of Biomechanics 40 (2007): 271–280, 10.1016/j.jbiomech.2006.01.007. 16524582

[54] H. E. Jaramillo , C. M. Puttlitz , K. McGilvray , and J. J. García , “Characterization of the L4–L5–S1 Motion Segment Using the Stepwise Reduction Method,” Journal of Biomechanics 49 (2016): 1248–1254, 10.1016/j.jbiomech.2016.02.050. 27017302

[55] H.‐J. Wilke , S. Grundler , C. Ottardi , C.‐E. Mathew , B. Schlager , and C. Liebsch , “In Vitro Analysis of Thoracic Spinal Motion Segment Flexibility During Stepwise Reduction of All Functional Structures,” European Spine Journal 29 (2020): 179–185, 10.1007/s00586-019-06196-7. 31664565

[56] H.‐J. Wilke , A. Herkommer , K. Werner , and C. Liebsch , “In Vitro Analysis of the Segmental Flexibility of the Thoracic Spine,” PLoS One 12 (2017): e0177823, 10.1371/journal.pone.0177823. 28520819 PMC5433776

[57] A. Beckmann , Biomechanical Investigation of Posterior Dynamic Stabilization Systems of the Lumbar Spine (RWTH Aachen University, 2021), 10.18154/RWTH-2021-05091.

[58] A. Beckmann , C. Herren , L. F. Nicolini , et al., “Biomechanical Testing of a Polycarbonate‐Urethane‐Based Dynamic Instrumentation System Under Physiological Conditions,” Clinical Biomechanics 61 (2019): 112–119, 10.1016/j.clinbiomech.2018.12.003. 30551087

[59] A. Beckmann , L. F. Nicolini , D. Grevenstein , et al., “Biomechanical In Vitro Test of a Novel Dynamic Spinal Stabilization System Incorporating Polycarbonate Urethane Material Under Physiological Conditions,” Journal of Biomechanical Engineering 142 (2020): 011005, 10.1115/1.4044242. 31314885

[60] L. F. Nicolini , R. C. Oliveira , M. Ribeiro , et al., “Tether Pretension Within Vertebral Body Tethering Reduces Motion of the Spine and Influences Coupled Motion: A Finite Element Analysis,” Computers in Biology and Medicine (2023): 107851. 38113683 10.1016/j.compbiomed.2023.107851

[61] P. Brinckmann and H. Grootenboer , “Change of Disc Height, Radial Disc Bulge, and Intradiscal Pressure From Discectomy an In Vitro Investigation on Human Lumbar Discs,” Spine 16 (1991): 641–646, 10.1097/00007632-199106000-00008. 1862403

[62] J. R. Meakin and D. W. L. Hukins , “Effect of Removing the Nucleus Pulposus on the Deformation of the Annulus Fibrosus During Compression of the Intervertebral Disc,” Journal of Biomechanics 33 (2000): 575–580, 10.1016/S0021-9290(99)00215-8. 10708778

[63] H. L. Guerin and D. M. Elliott , “Quantifying the Contributions of Structure to Annulus Fibrosus Mechanical Function Using a Nonlinear, Anisotropic, Hyperelastic Model,” Journal of Orthopaedic Research 25 (2007): 508–516, 10.1002/jor.20324. 17149747

[64] T. Tsai , C. Cheng , C. Chen , and P. Lai , “Mechanotransduction in Intervertebral Discs,” Journal of Cellular and Molecular Medicine 18 (2014): 2351–2360, 10.1111/jcmm.12377. 25267492 PMC4302640

[65] K. Sato , S. Kikuchi , and T. Yonezawa , “In Vivo Intradiscal Pressure Measurement in Healthy Individuals and in Patients With Ongoing Back Problems,” Spine 24 (1999): 2468–2474, 10.1097/00007632-199912010-00008. 10626309

[66] F. Heuer , H. Schmidt , L. Claes , and H.‐J. Wilke , “Stepwise Reduction of Functional Spinal Structures Increase Vertebral Translation and Intradiscal Pressure,” Journal of Biomechanics 40 (2007): 795–803. 16712856 10.1016/j.jbiomech.2006.03.016

[67] A. Rohlmann , L. Bauer , T. Zander , G. Bergmann , and H.‐J. Wilke , “Determination of Trunk Muscle Forces for Flexion and Extension by Using a Validated Finite Element Model of the Lumbar Spine and Measured In Vivo Data,” Journal of Biomechanics 39 (2006): 981–989, 10.1016/j.jbiomech.2005.02.019. 16549091

[68] L. F. Nicolini , The Effects of Vertebral Body Tethering System on the Biomechanics of the Thoracolumbar Spine (Federal University of Santa Catarina and RWTH Aachen University, 2023), 10.18154/RWTH-2023-04766.

[69] J. H. Kim , A. Sharan , W. Cho , M. Emam , M. Hagen , and S. Y. Kim , “The Prevalence of Asymptomatic Cervical and Lumbar Facet Arthropathy: A Computed Tomography Study,” Asian Spine Journal 13 (2019): 417–422, 10.31616/asj.2018.0235. 30744307 PMC6547401

[70] R. A. Tisot , J. d. S. Vieira , D. d. S. Collares , et al., “Facet Joint Degeneration in Patients With Lumbar Disc Herniation and Probable Determining Factors,” Coluna/Columna 19 (2020): 262–265, 10.1590/S1808-185120201904222827.

[71] X. Lv , Y. Liu , S. Zhou , et al., “Correlations Between the Feature of Sagittal Spinopelvic Alignment and Facet Joint Degeneration: A Retrospective Study,” BMC Musculoskeletal Disorders 17 (2016): 1–5, 10.1186/s12891-016-1193-6. 27528107 PMC4986370

[72] M. Dreischarf , T. Zander , A. Shirazi‐Adl , et al., “Comparison of Eight Published Static Finite Element Models of the Intact Lumbar Spine: Predictive Power of Models Improves When Combined Together,” Journal of Biomechanics 47 (2014): 1757–1766, 10.1016/j.jbiomech.2014.04.002. 24767702

[73] M. Nikkhoo , M.‐L. Lu , W.‐C. Chen , et al., “Biomechanical Investigation Between Rigid and Semirigid Posterolateral Fixation During Daily Activities: Geometrically Parametric Poroelastic Finite Element Analyses,” Frontiers in Bioengineering and Biotechnology 9 (2021): 646079, 10.3389/fbioe.2021.646079. 33869156 PMC8047206

[74] B. A. Jamjoom , S. Patel , R. Bommireddy , and Z. Klezl , “Impact of the Quantity of Intradiscal Cement Leak on the Progression of Intervertebral Disc Degeneration,” Annals of the Royal College of Surgeons of England 99 (2017): 529–533, 10.1308/rcsann.2017.0083. 28853606 PMC5697037

[75] M. Rohanifar , S. W. Clayton , G. W. D. Easson , et al., “Single Cell RNA‐Sequence Analyses Reveal Uniquely Expressed Genes and Heterogeneous Immune Cell Involvement in the Rat Model of Intervertebral Disc Degeneration,” Applied Sciences 12 (2022): 1–26, 10.3390/app12168244. PMC9706593 36451894

[76] C. Feng , M. Yang , Y. Zhang , et al., “Cyclic Mechanical Tension Reinforces DNA Damage and Activates the p53‐p21‐Rb Pathway to Induce Premature Senescence of Nucleus Pulposus Cells,” International Journal of Molecular Medicine 41 (2018): 3316–3326, 10.3892/ijmm.2018.3522. 29512682 PMC5881642

[77] A. Yucekul , B. Akpunarli , A. Durbas , et al., “Does Vertebral Body Tethering Cause Disc and Facet Joint Degeneration? A Preliminary MRI Study With Minimum Two Years Follow‐Up,” Spine Journal 21 (2021): 1793–1801, 10.1016/j.spinee.2021.05.020. 34033932

