# Supplementary material for: The Effects of Vertebral Body Tethering on the Intervertebral Discs and Facet Joints: A Numerical Analysis
Source: Int J Numer Method Biomed Eng. 2025 Aug 11;41(8):e70084. doi: 10.1002/cnm.70084 (PMC12339148; doi:10.1002/cnm.70084)
Supplement: Supplementary file 1 — Data S1: Supporting Information. [file CNM-41-e70084-s001.pdf]

SUPPLEMENTARY MATERIAL

The material properties of the intervertebral disc of model L1-L2 are shown in Table 1.

Table 1 Parameter values representing the material properties of the L1-L2 intervertebral disc. The coefficients k_{1c}, k_{2c} and β_c were defined to generate a circumferential gradient in the values of k_1, k_2 and β along the perimeter of the annulus fibrosus, respectively. Similarly, k_{1r} and k_{2r}were assigned to control the variation of k_1 and k_2 along the radial direction of the annulus fibrosus [1]. Source: Nicolini et al. [2].

Nucleus pulposus			Annulus fibrosus										
C_{10n}	C_{01n}	D_n	C_{10}	D	k_1	k_2	k	β	k_{1c}	k_{2c}	k_{1r}	k_{2r}	β_c
0.03	0.19	0	0.22	0	4.5	300	0.1	30	-0.05	-0.05	-0.15	-0.15	0.1

Tie constraints were used to attach anchors, screAws and intervertebral discs to the vertebral bodies as shown in Fig. 1.

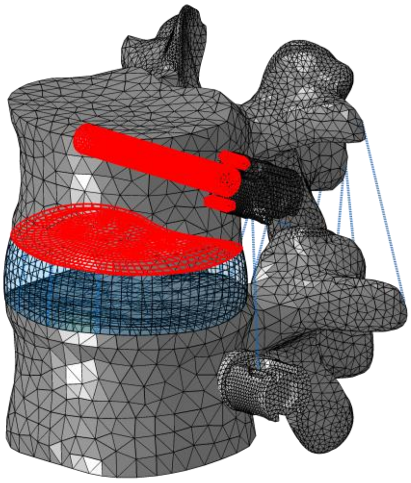

Fig. 1 Perspective view of part of the finite element model. Tie constraints (red part) was used to attach anchors and screws to the vertebral bodies as well as at the interface between the disc and vertebral body endplate.

The below figures show the comparison between the numerical and experimental results of the spine from literature [1,3–12] for various loading directions and segments. The legend "Beckmann et al. (2021)T" and "Beckmann et al. (2021)MP" refers to the Transition Study and Move-P Study with experimental data obtained from Beckmann et al. [10].

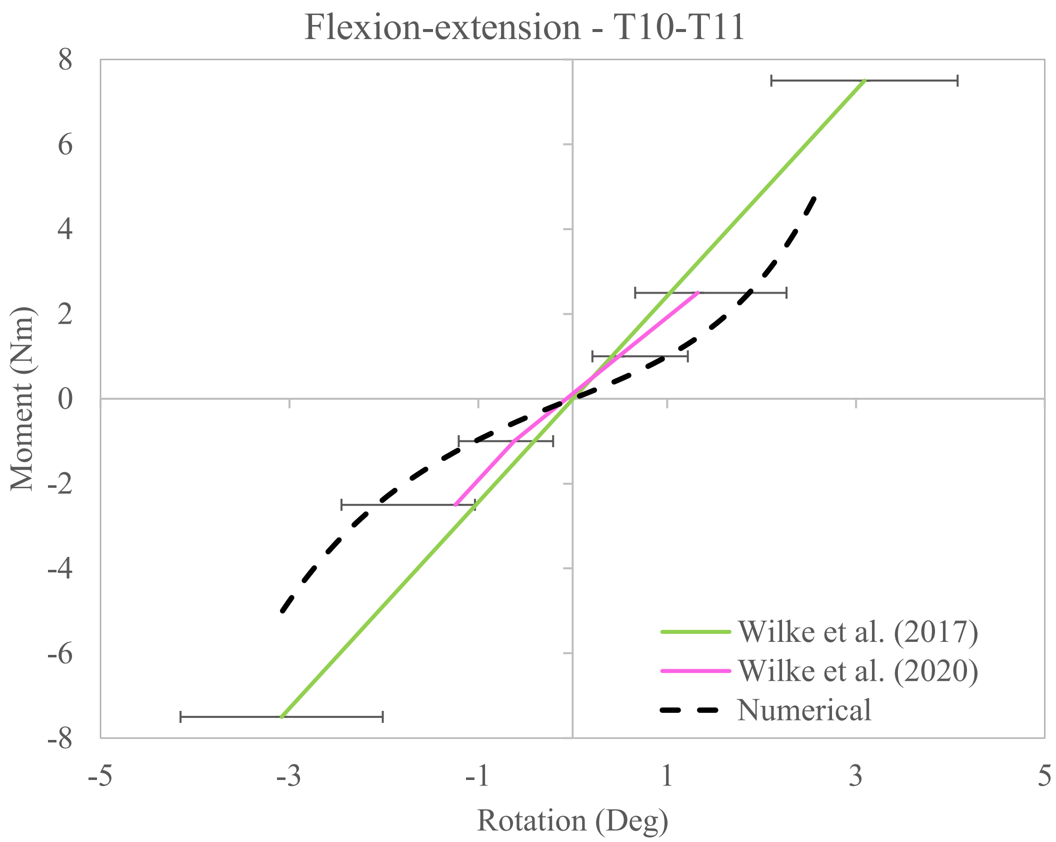

Flexion-extension - T10-T11
Moment (Nm)
8
6
4
2
0
-2
-4
-6
-8
-5
-3
-1
1
3
5
Rotation (Deg)
Wilke et al. (2017)
Wilke et al. (2020)
Numerical

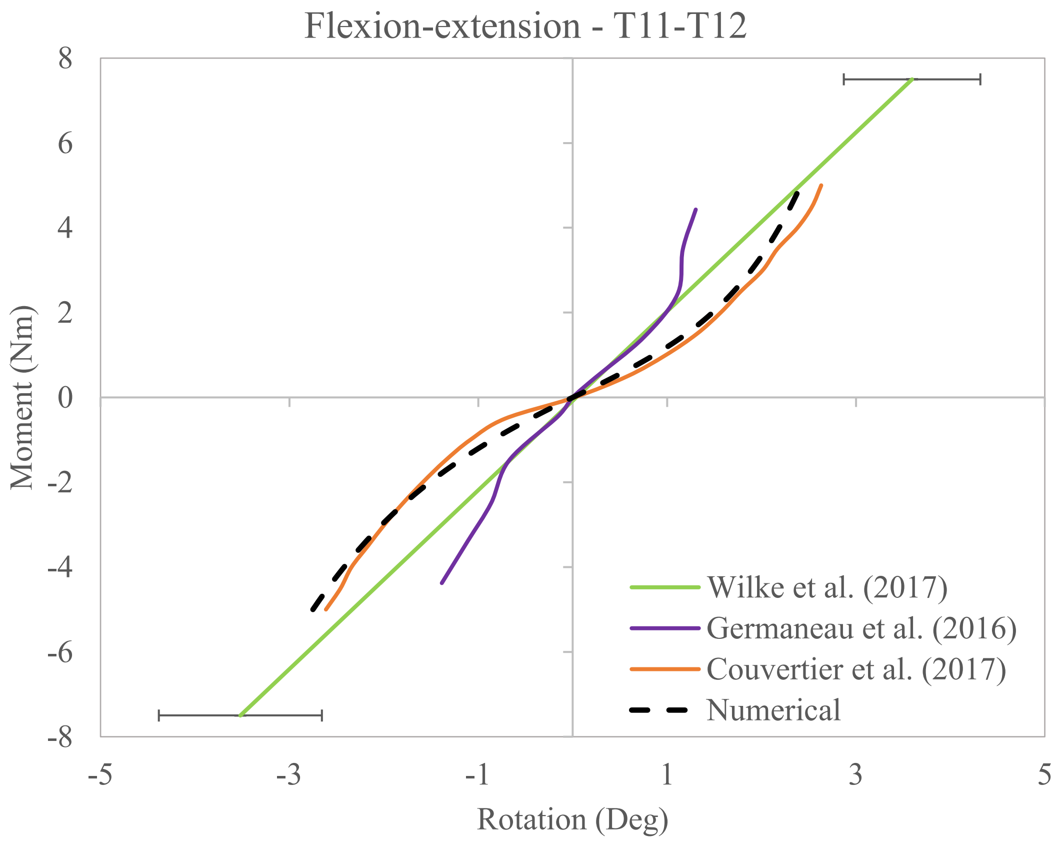

Flexion-extension - T11-T12
Moment (Nm)
8
6
4
2
0
-2
-4
-6
-8
-5
-3
-1
1
3
5
Rotation (Deg)
Wilke et al. (2017)
Germaneau et al. (2016)
Couvertier et al. (2017)
Numerical

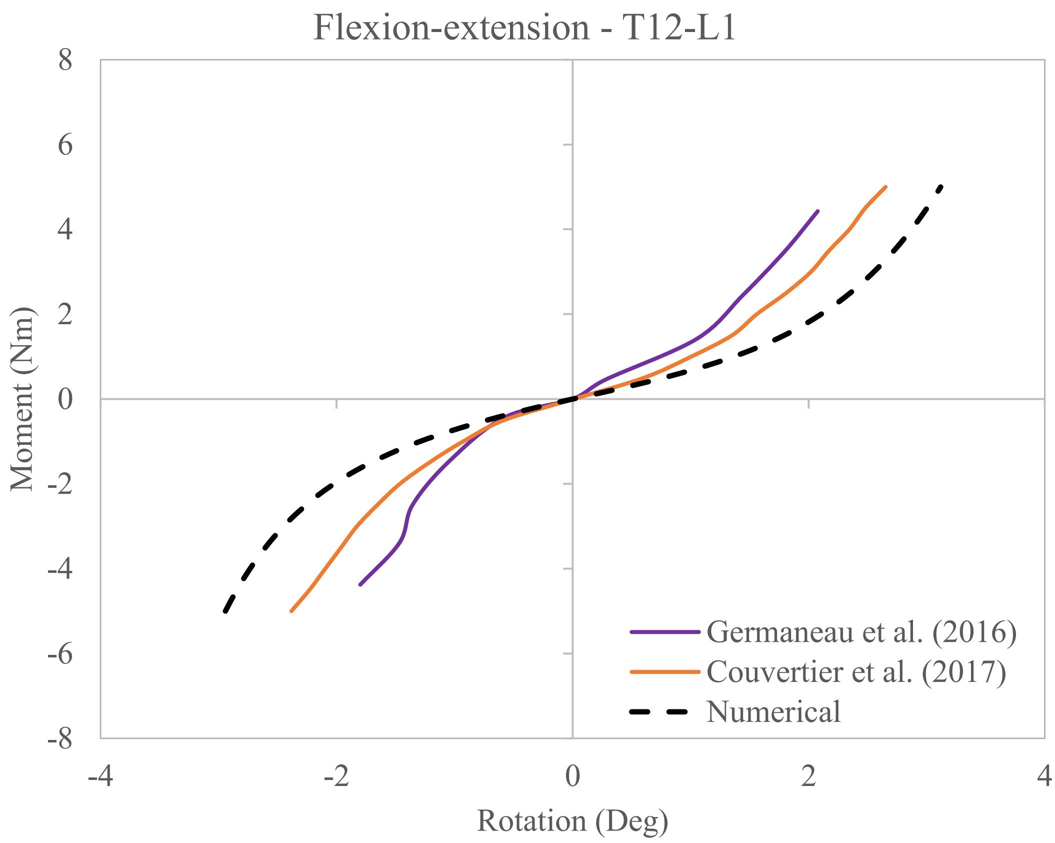

Flexion-extension - T12-L1
Moment (Nm)
Rotation (Deg)
8
6
4
2
0
-2
-4
-6
-8
-4
-2
0
2
4
Germaneau et al. (2016)
Couvertier et al. (2017)
Numerical

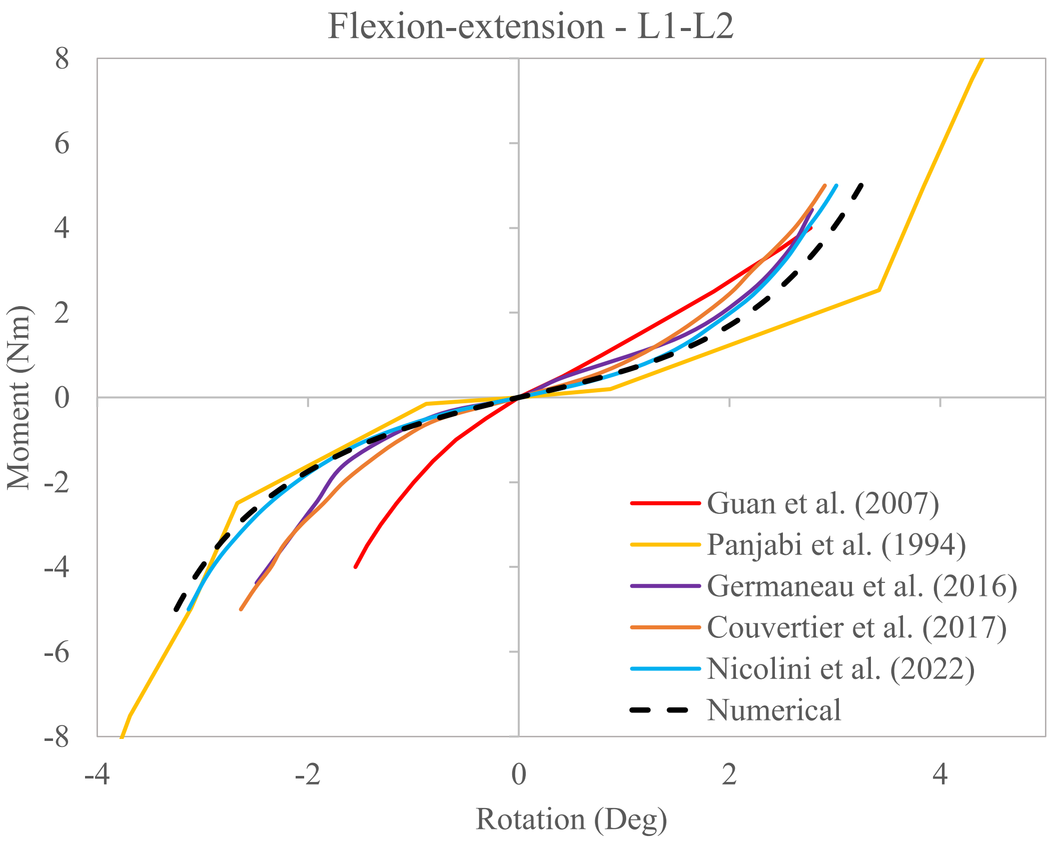

Flexion-extension - L1-L2
Moment (Nm)
Rotation (Deg)
8
6
4
2
0
-2
-4
-6
-8
-4
-2
0
2
4
Guan et al. (2007)
Panjabi et al. (1994)
Germaneau et al. (2016)
Couvertier et al. (2017)
Nicolini et al. (2022)
Numerical

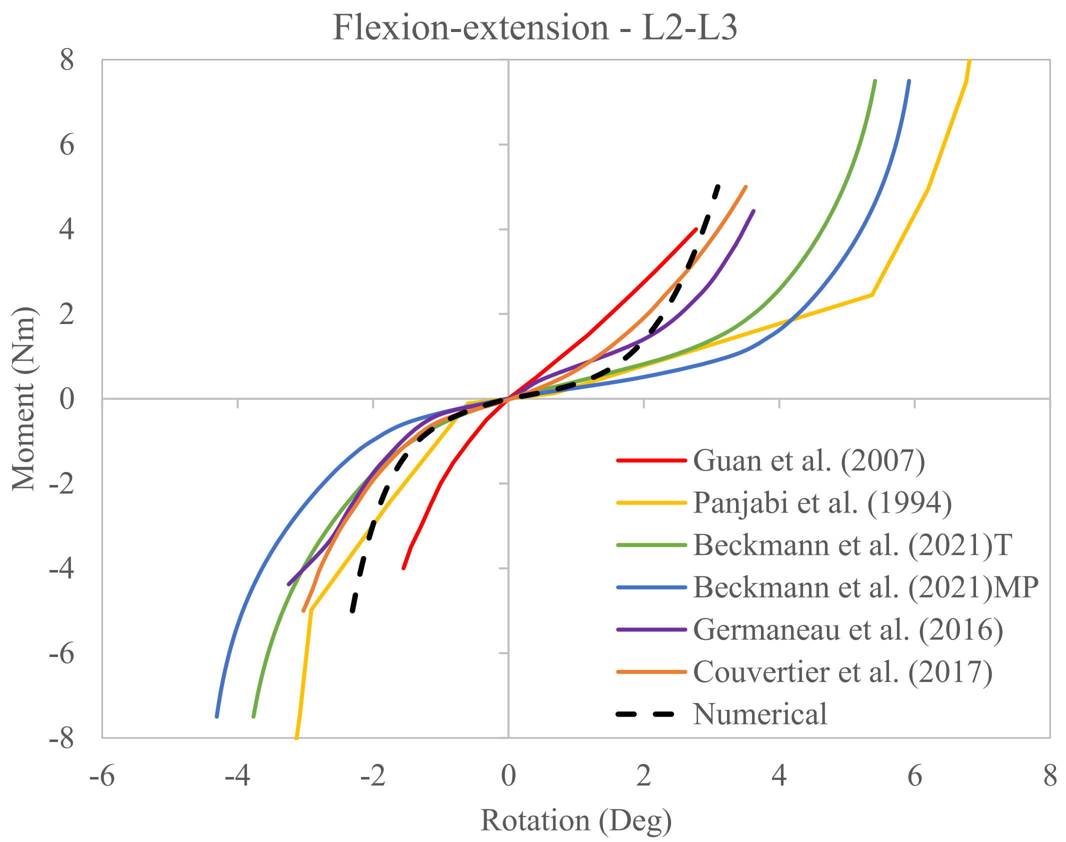
Flexion-extension - L2-L3
Moment (Nm)
Rotation (Deg)
8
6
4
2
0
-2
-4
-6
-8
-6
-4
-2
0
2
4
6
8
Guan et al. (2007)
Panjabi et al. (1994)
Beckmann et al. (2021)T
Beckmann et al. (2021)MP
Germaneau et al. (2016)
Couvertier et al. (2017)
Numerical

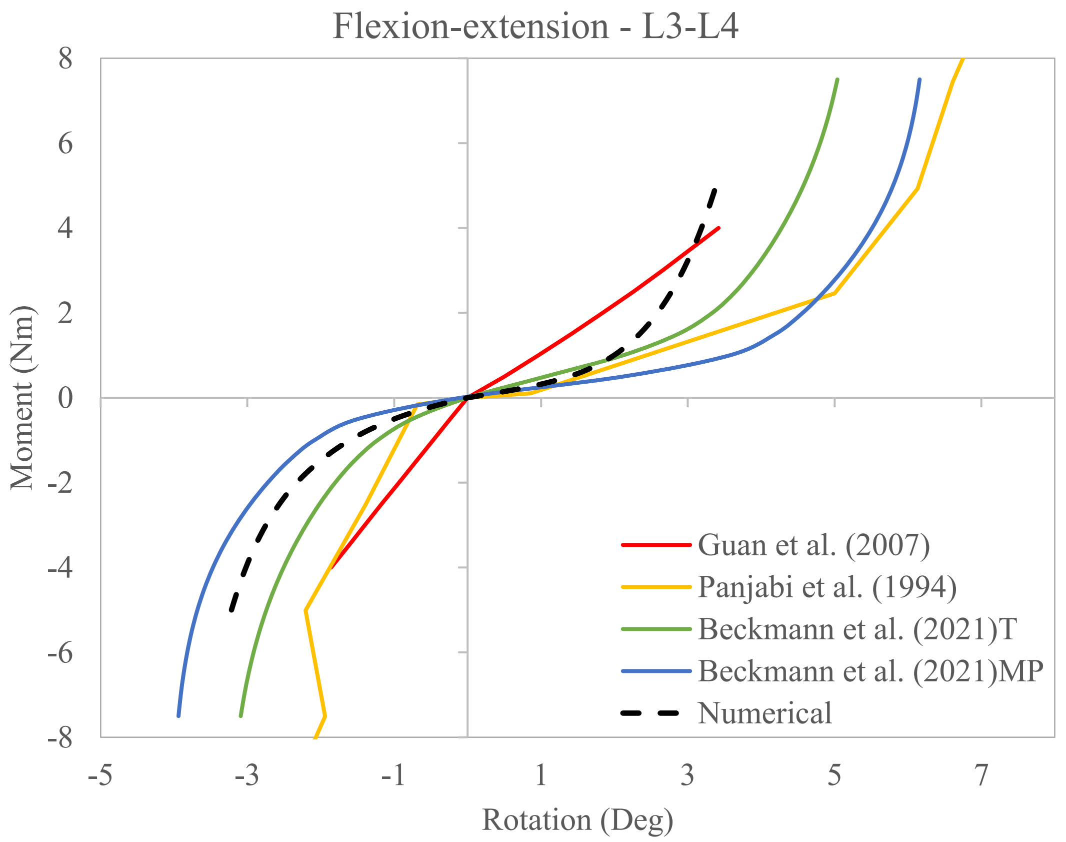
Flexion-extension - L3-L4
Moment (Nm)
Rotation (Deg)
8
6
4
2
0
-2
-4
-6
-8
-5
-3
-1
1
3
5
7
Guan et al. (2007)
Panjabi et al. (1994)
Beckmann et al. (2021)T
Beckmann et al. (2021)MP
Numerical

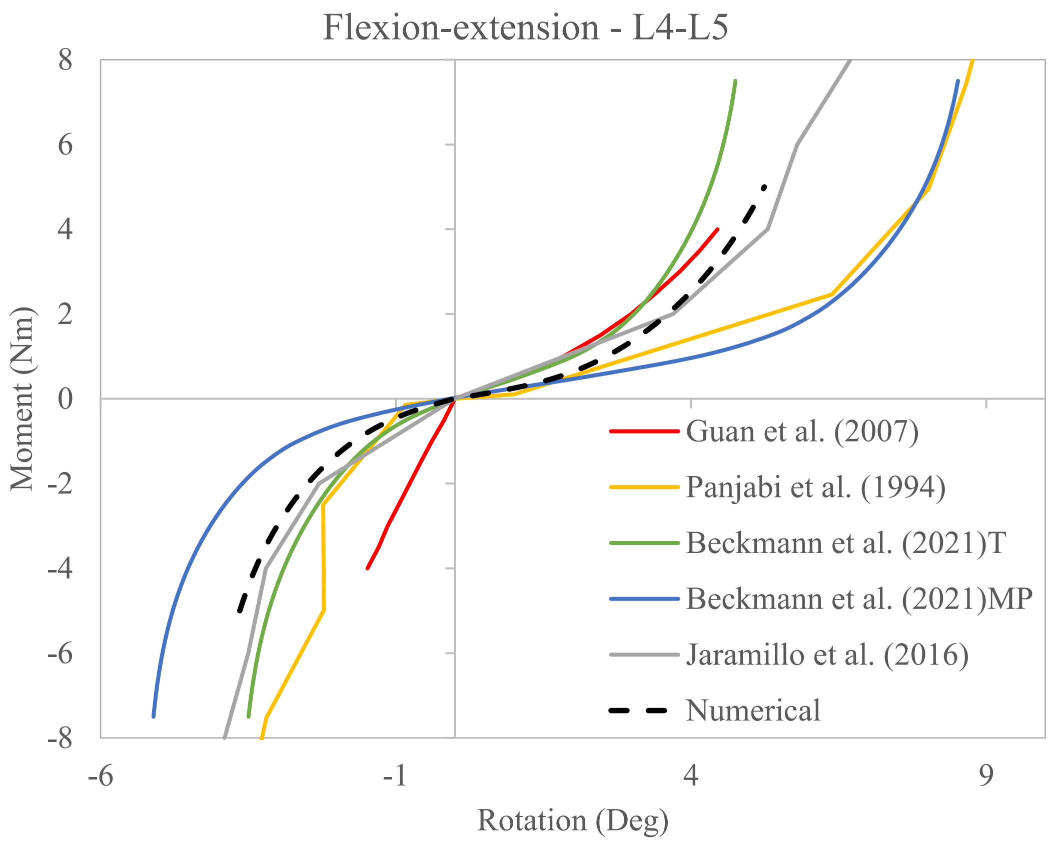
Flexion-extension - L4-L5
Moment (Nm)
Rotation (Deg)
Guan et al. (2007)
Panjabi et al. (1994)
Beckmann et al. (2021)T
Beckmann et al. (2021)MP
Jaramillo et al. (2016)
Numerical

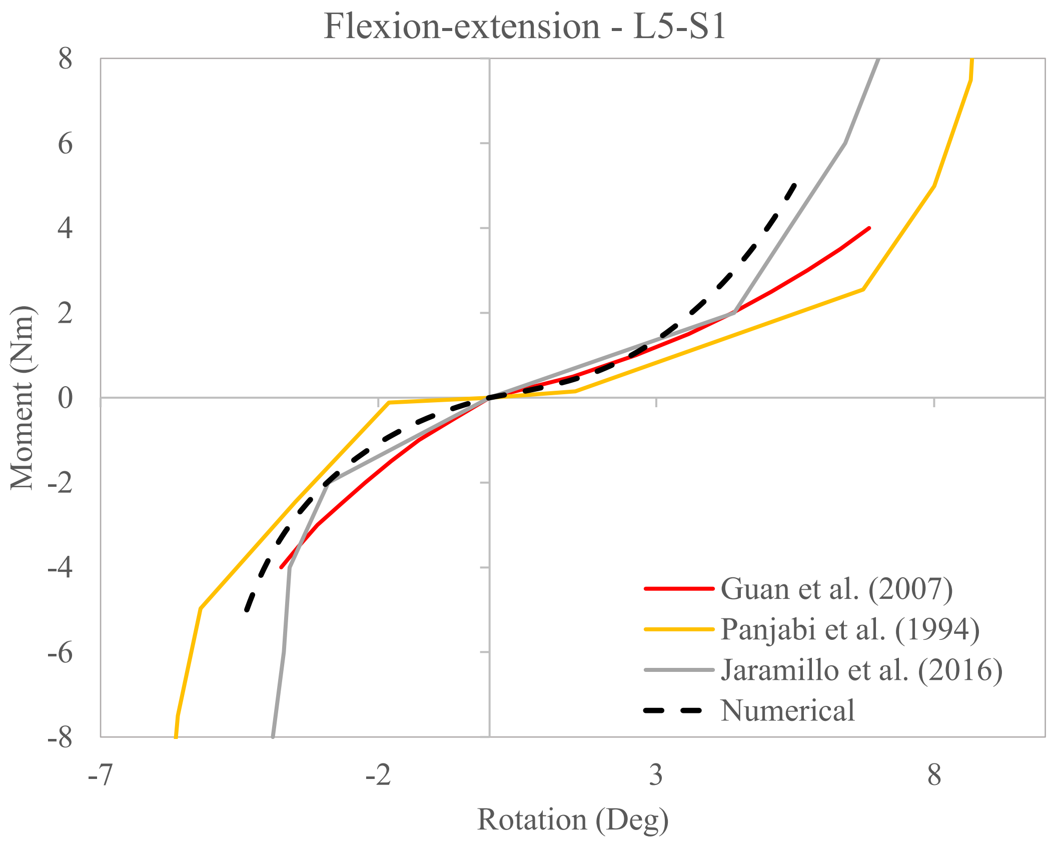
Flexion-extension - L5-S1
Moment (Nm)
Rotation (Deg)
Guan et al. (2007)
Panjabi et al. (1994)
Jaramillo et al. (2016)
Numerical

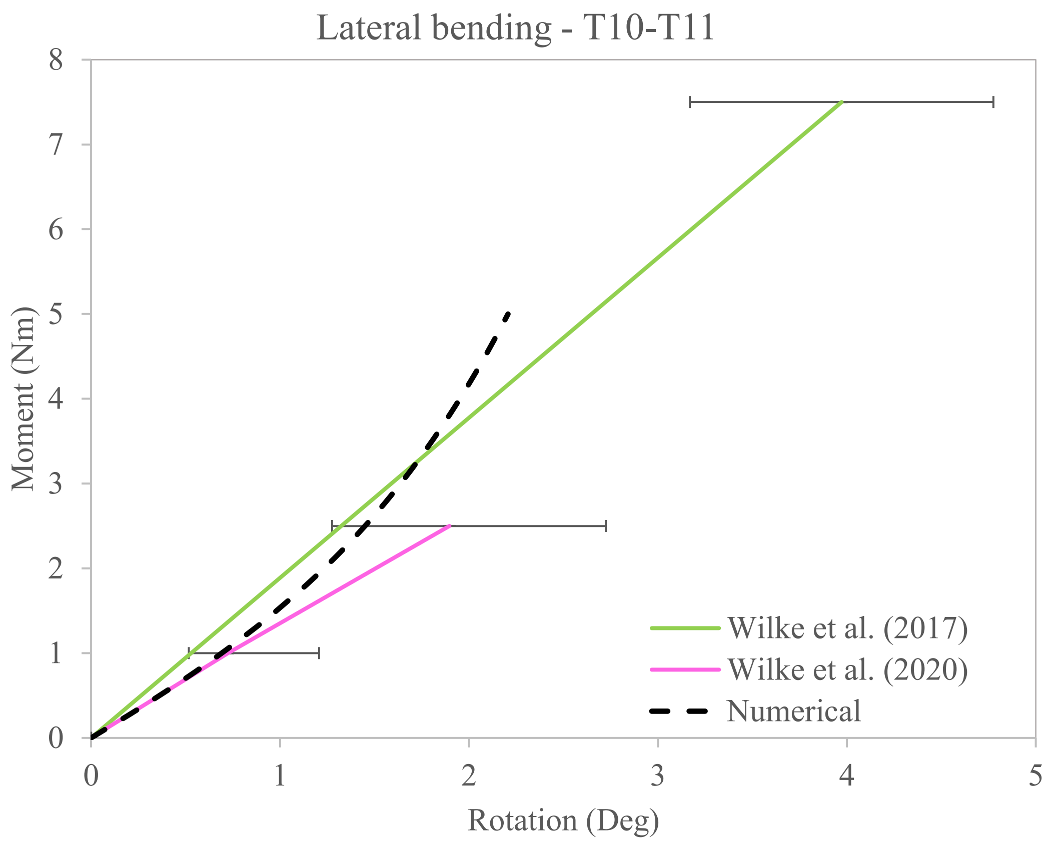
Lateral bending - T10-T11
Moment (Nm)
Rotation (Deg)
0
1
2
3
4
5
6
7
8
0
1
2
3
4
5
Wilke et al. (2017)
Wilke et al. (2020)
Numerical

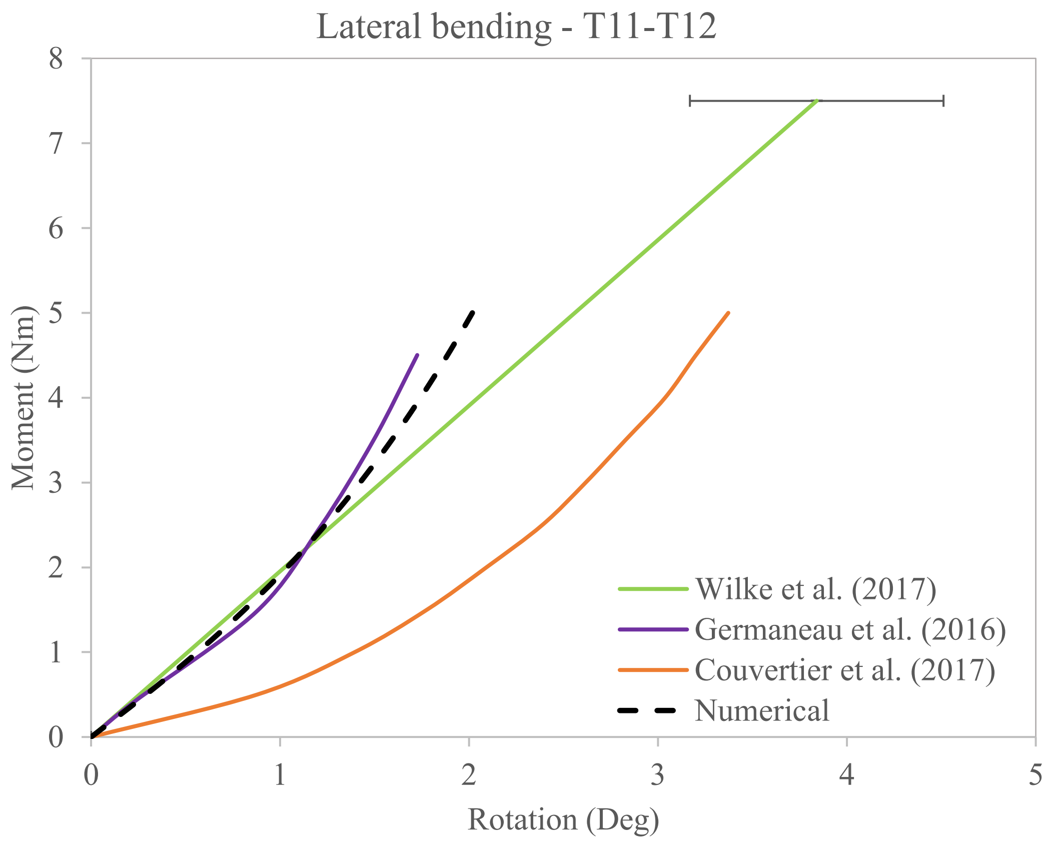
Lateral bending - T11-T12
Moment (Nm)
Rotation (Deg)
0
1
2
3
4
5
6
7
8
0
1
2
3
4
5
Wilke et al. (2017)
Germaneau et al. (2016)
Couvertier et al. (2017)
Numerical

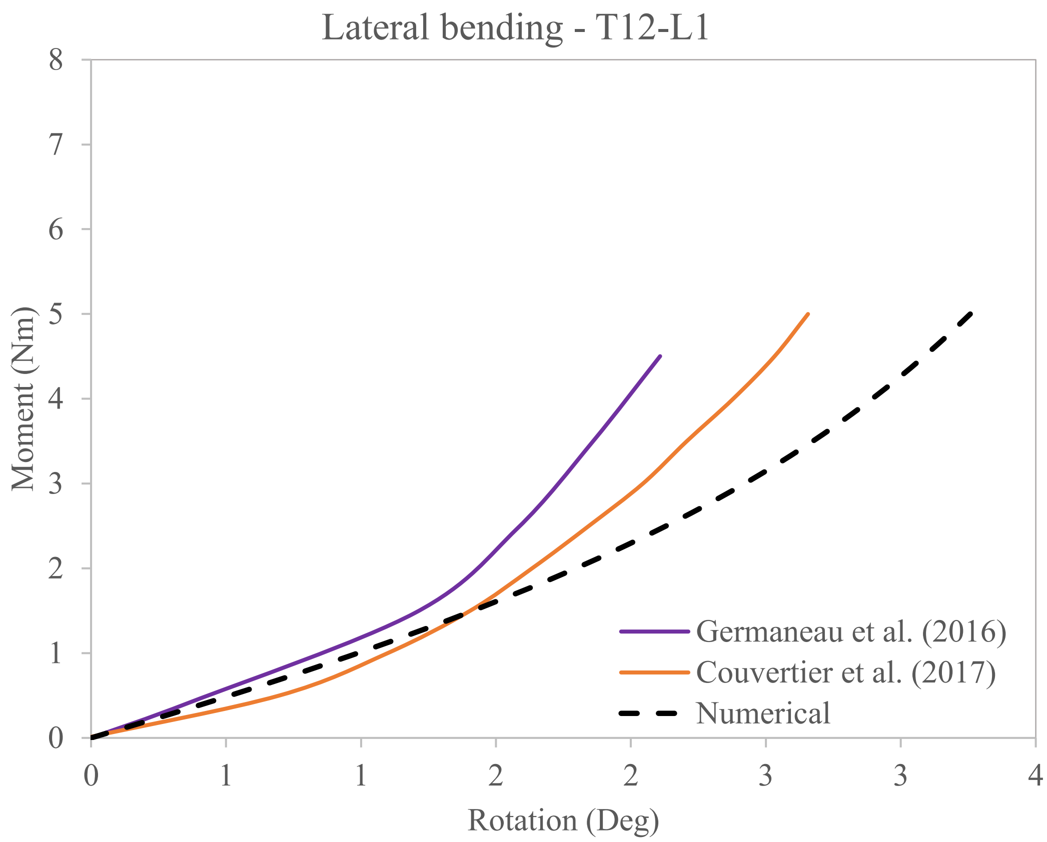
Lateral bending - T12-L1
Moment (Nm)
Rotation (Deg)
0
1
2
3
4
5
6
7
8
0
1
1
2
2
3
3
4
Germaneau et al. (2016)
Couvertier et al. (2017)
Numerical

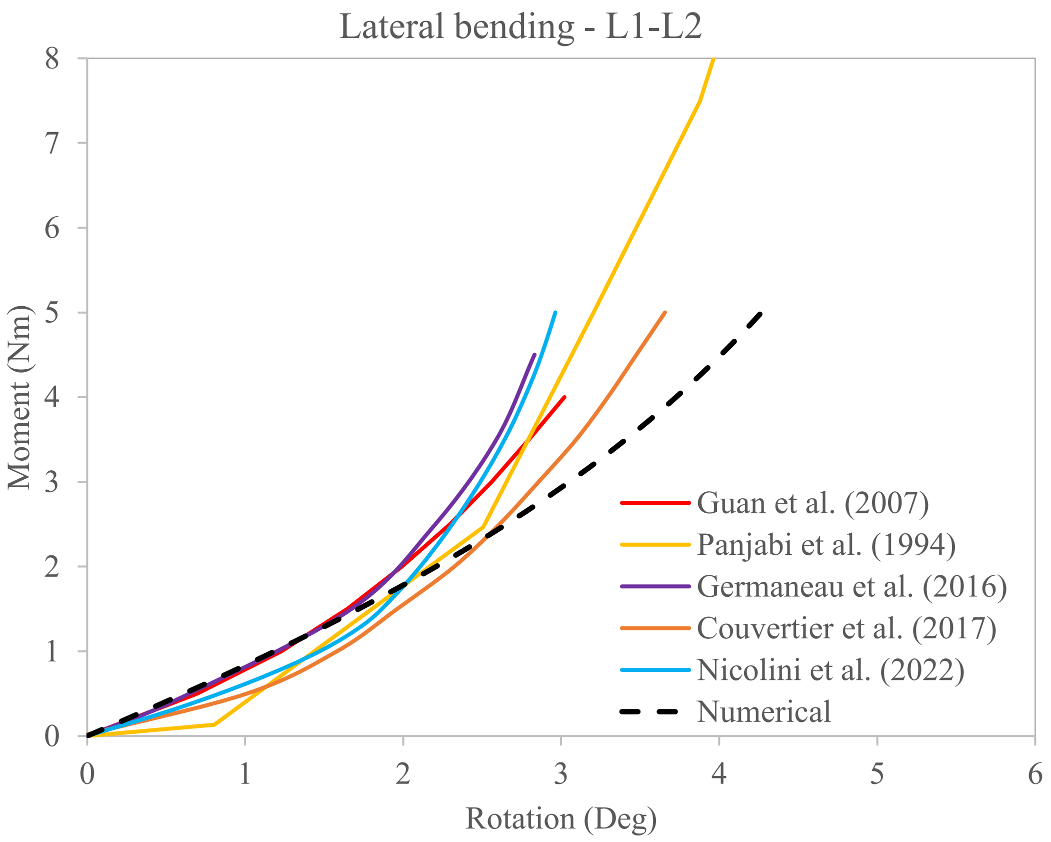
Lateral bending - L1-L2
Moment (Nm)
Rotation (Deg)
0
1
2
3
4
5
6
7
8
0
1
2
3
4
5
6
Guan et al. (2007)
Panjabi et al. (1994)
Germaneau et al. (2016)
Couvertier et al. (2017)
Nicolini et al. (2022)
Numerical

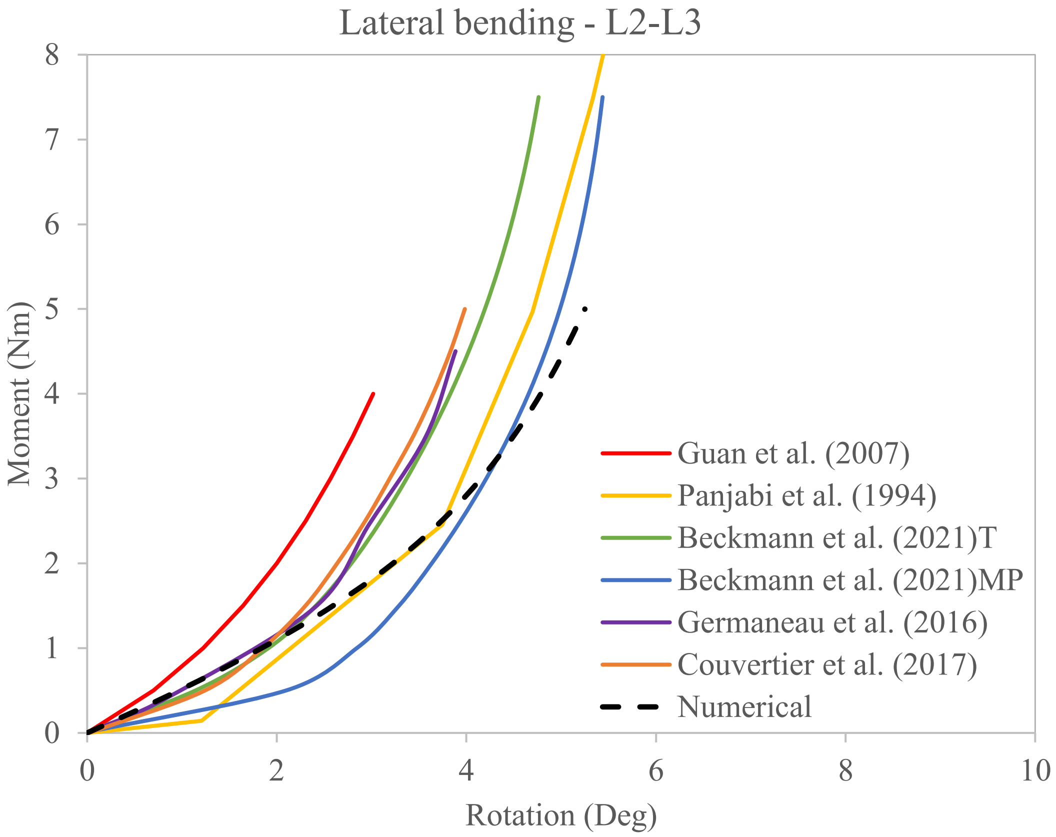

Lateral bending - L2-L3
Moment (Nm)
Rotation (Deg)
0
1
2
3
4
5
6
7
8
0
2
4
6
8
10
Guan et al. (2007)
Panjabi et al. (1994)
Beckmann et al. (2021)T
Beckmann et al. (2021)MP
Germaneau et al. (2016)
Couvertier et al. (2017)
Numerical

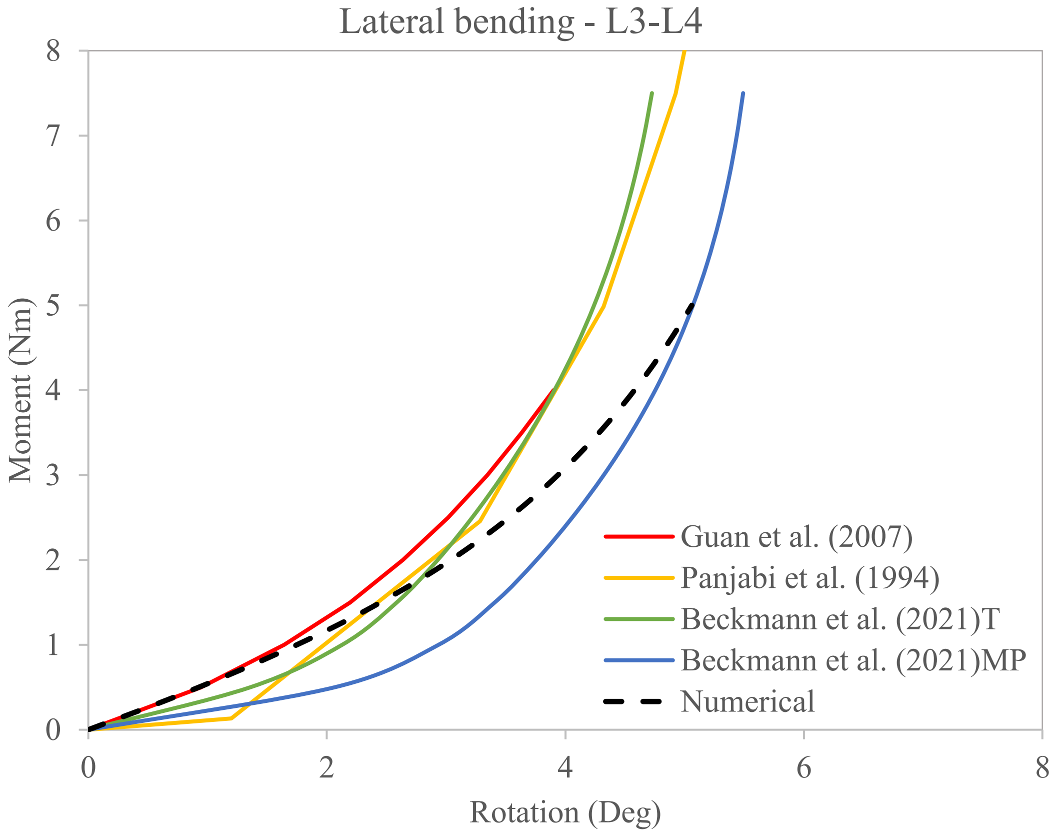

Lateral bending - L3-L4
Moment (Nm)
Rotation (Deg)
0
1
2
3
4
5
6
7
8
0
2
4
6
8
Guan et al. (2007)
Panjabi et al. (1994)
Beckmann et al. (2021)T
Beckmann et al. (2021)MP
Numerical

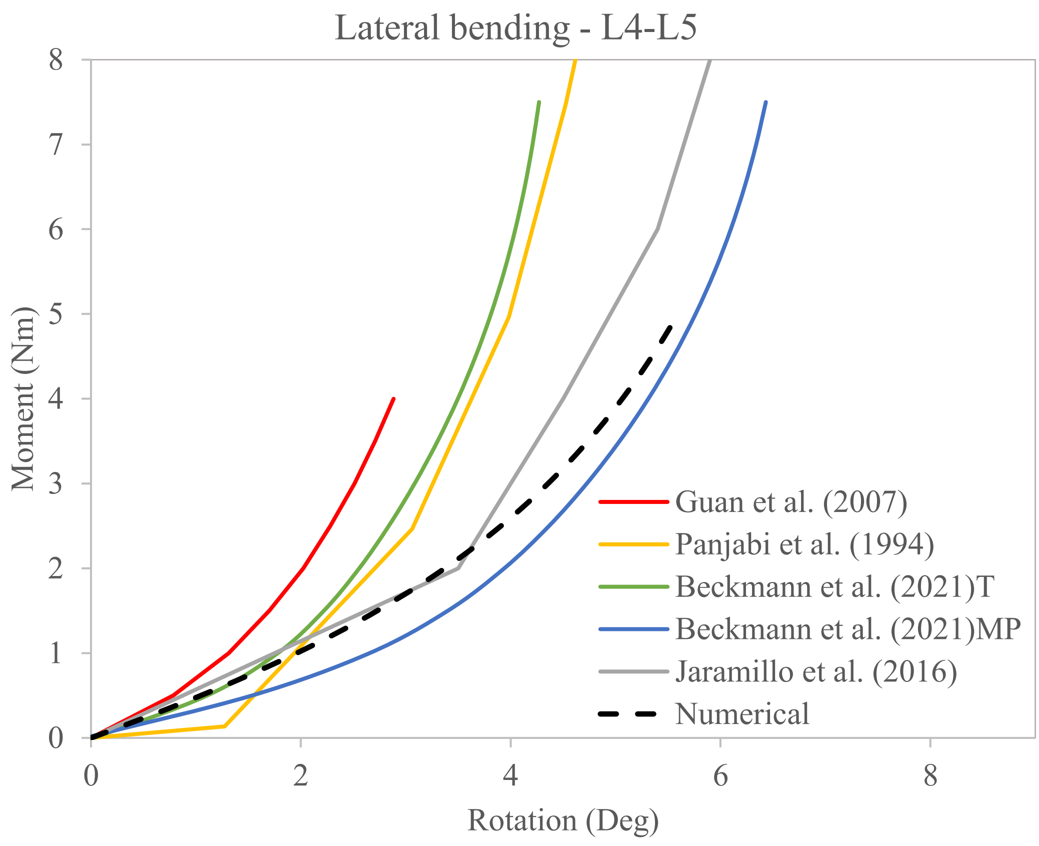

Lateral bending - L4-L5
Moment (Nm)
0
1
2
3
4
5
6
7
8
0
2
4
6
8
Rotation (Deg)
Guan et al. (2007)
Panjabi et al. (1994)
Beckmann et al. (2021)T
Beckmann et al. (2021)MP
Jaramillo et al. (2016)
Numerical

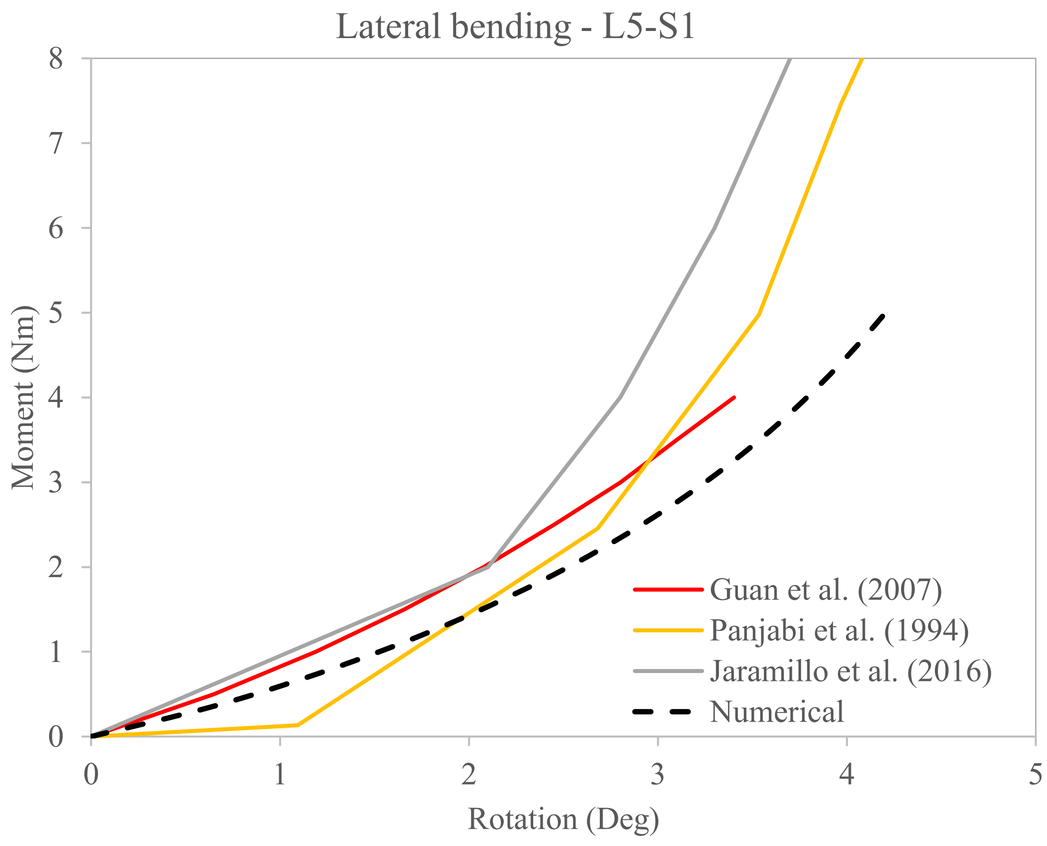

Lateral bending - L5-S1
Moment (Nm)
0
1
2
3
4
5
6
7
8
0
1
2
3
4
5
Rotation (Deg)
Guan et al. (2007)
Panjabi et al. (1994)
Jaramillo et al. (2016)
Numerical

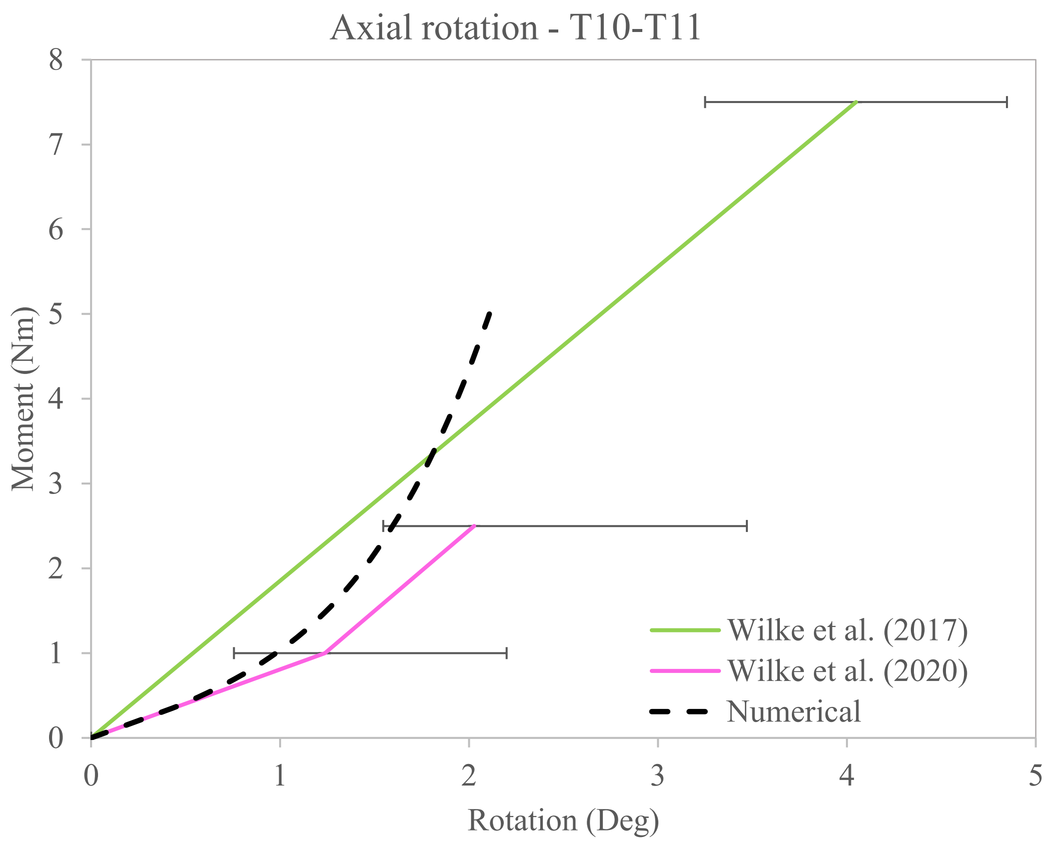

Axial rotation - T10-T11
Moment (Nm)
Rotation (Deg)
Wilke et al. (2017)
Wilke et al. (2020)
Numerical

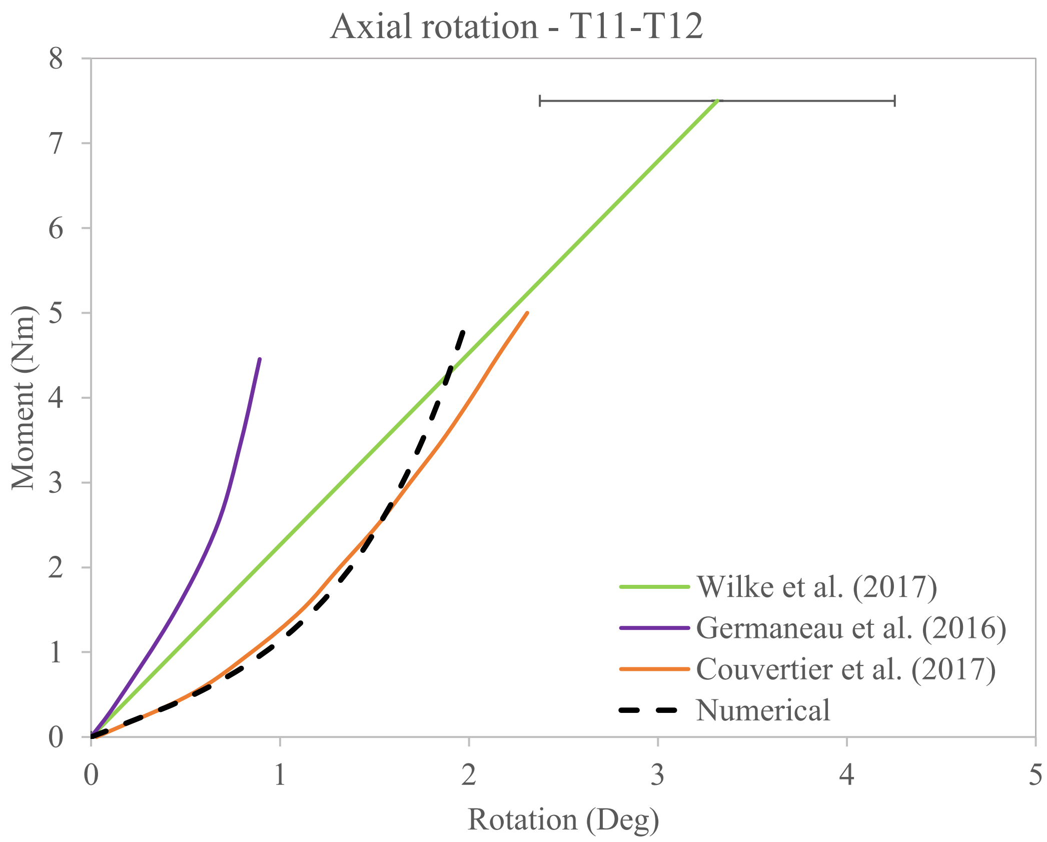

Axial rotation - T11-T12
Moment (Nm)
Rotation (Deg)
Wilke et al. (2017)
Germaneau et al. (2016)
Couvertier et al. (2017)
Numerical

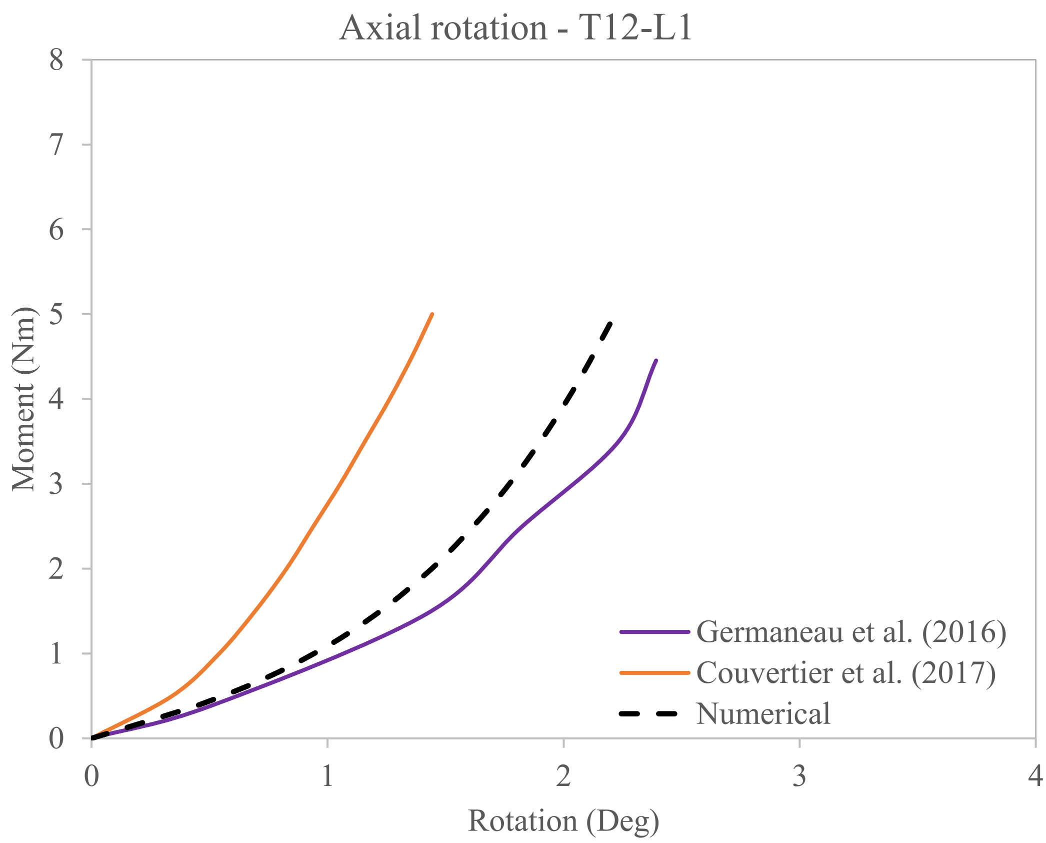
Axial rotation - T12-L1
Moment (Nm)
Rotation (Deg)
0
1
2
3
4
5
6
7
8
0
1
2
3
4
Germaneau et al. (2016)
Couvertier et al. (2017)
Numerical

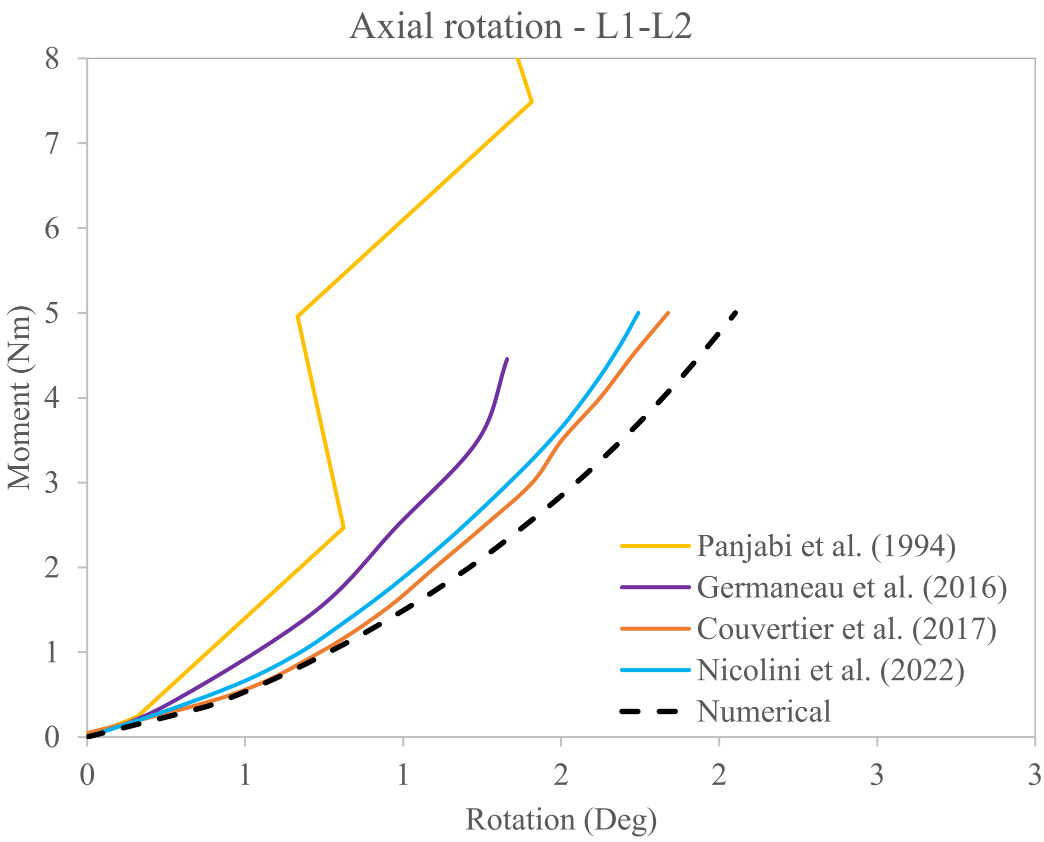
Axial rotation - L1-L2
Moment (Nm)
Rotation (Deg)
0
1
2
3
4
5
6
7
8
0
1
1
2
2
3
3
Panjabi et al. (1994)
Germaneau et al. (2016)
Couvertier et al. (2017)
Nicolini et al. (2022)
Numerical

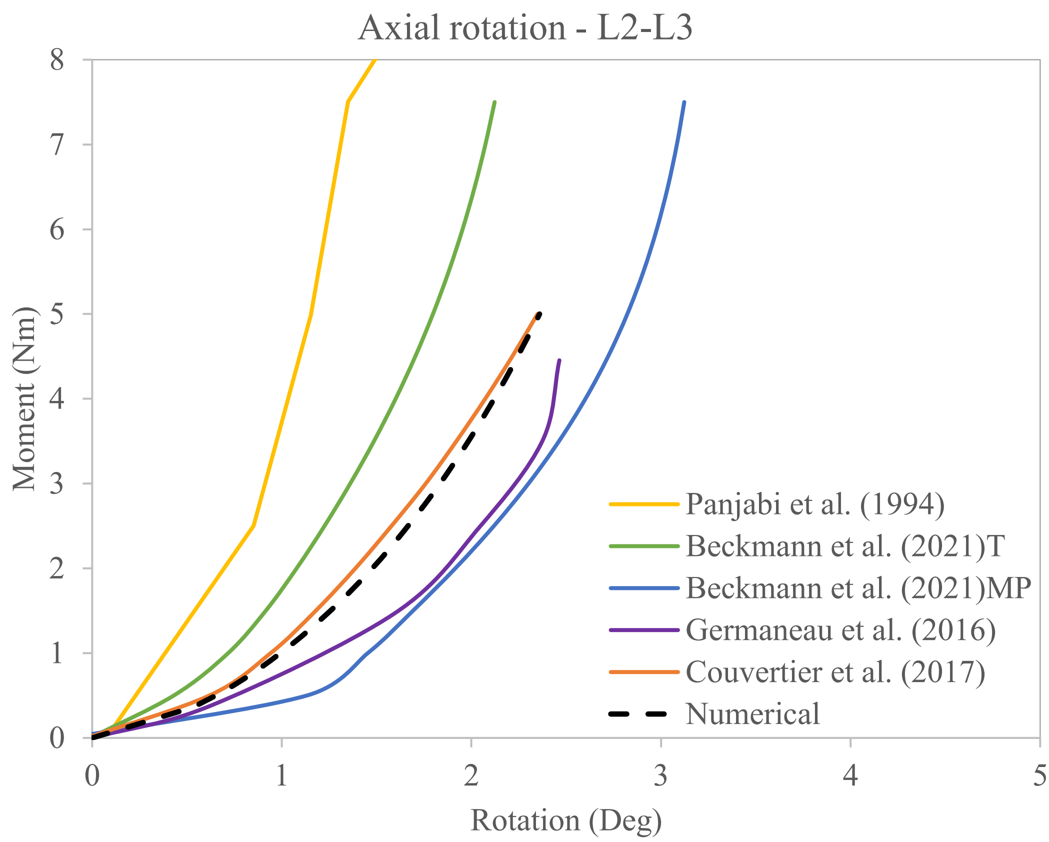
Axial rotation - L2-L3
Moment (Nm)
Rotation (Deg)
0
1
2
3
4
5
6
7
8
0
1
2
3
4
5
Panjabi et al. (1994)
Beckmann et al. (2021)T
Beckmann et al. (2021)MP
Germaneau et al. (2016)
Couvertier et al. (2017)
Numerical

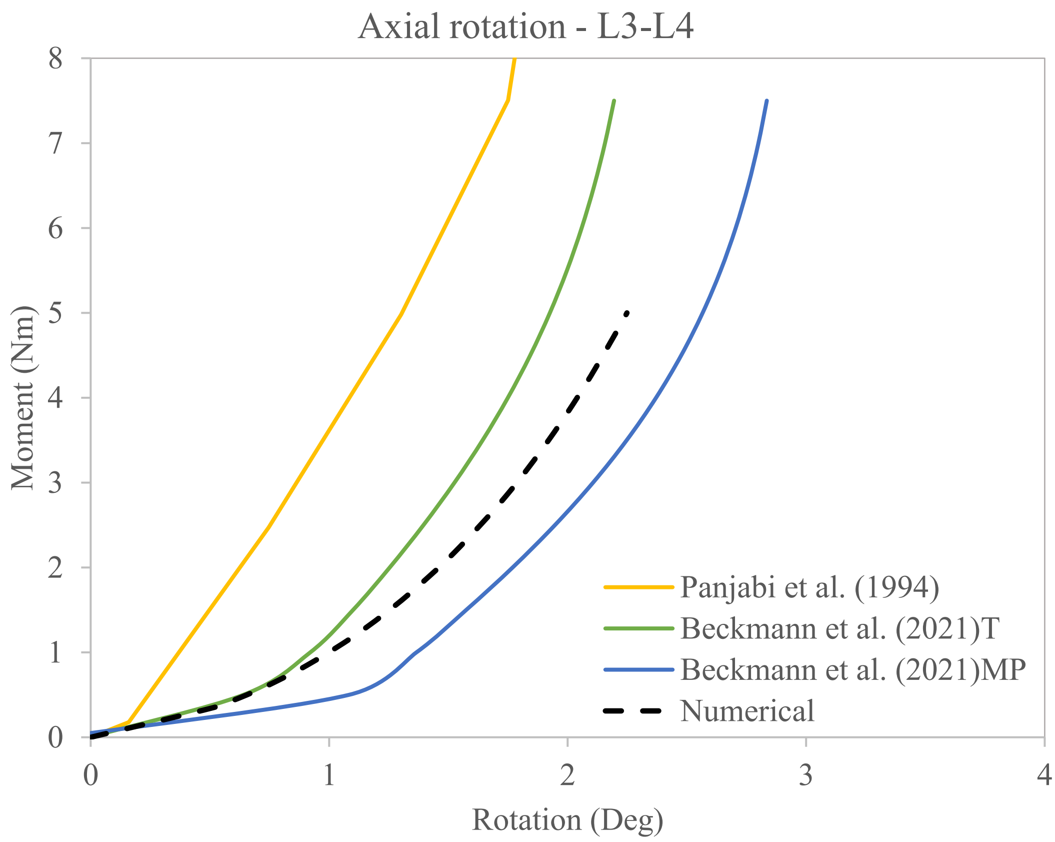
Axial rotation - L3-L4
Moment (Nm)
Rotation (Deg)
0
1
2
3
4
5
6
7
8
0
1
2
3
4
Panjabi et al. (1994)
Beckmann et al. (2021)T
Beckmann et al. (2021)MP
Numerical

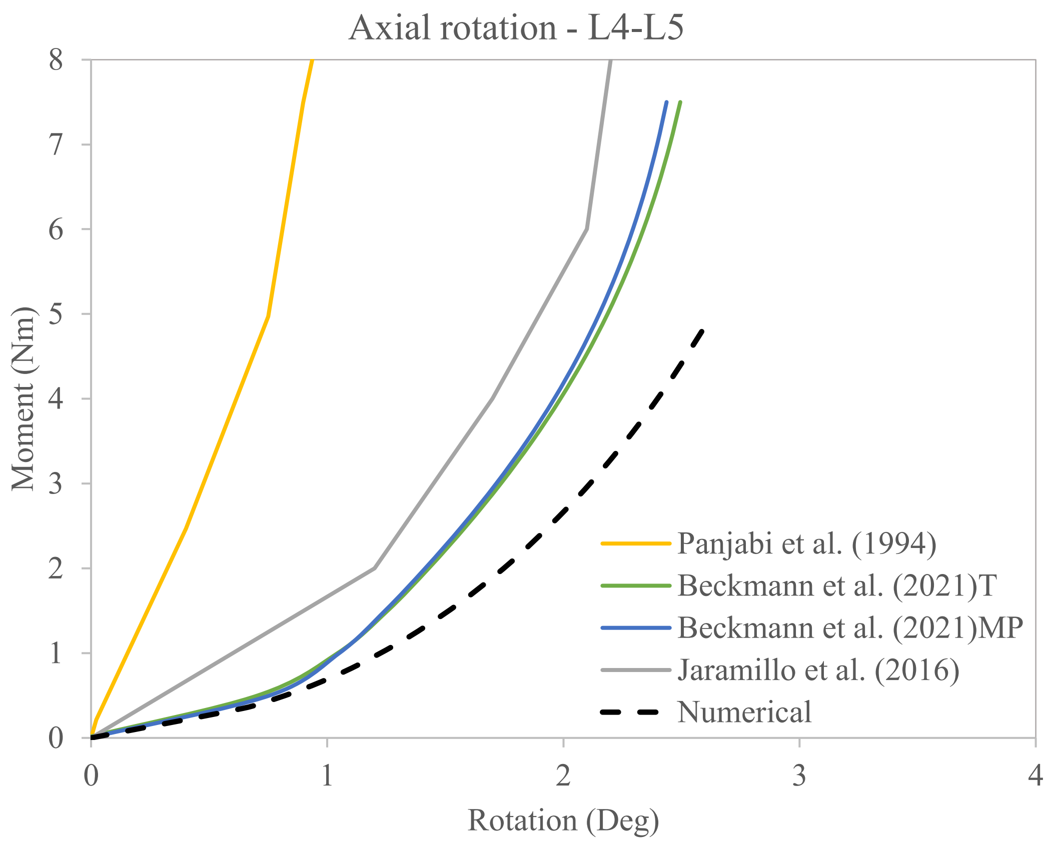
Axial rotation - L4-L5
Moment (Nm)
Rotation (Deg)
0
1
2
3
4
5
6
7
8
0
1
2
3
4
Panjabi et al. (1994)
Beckmann et al. (2021)T
Beckmann et al. (2021)MP
Jaramillo et al. (2016)
Numerical

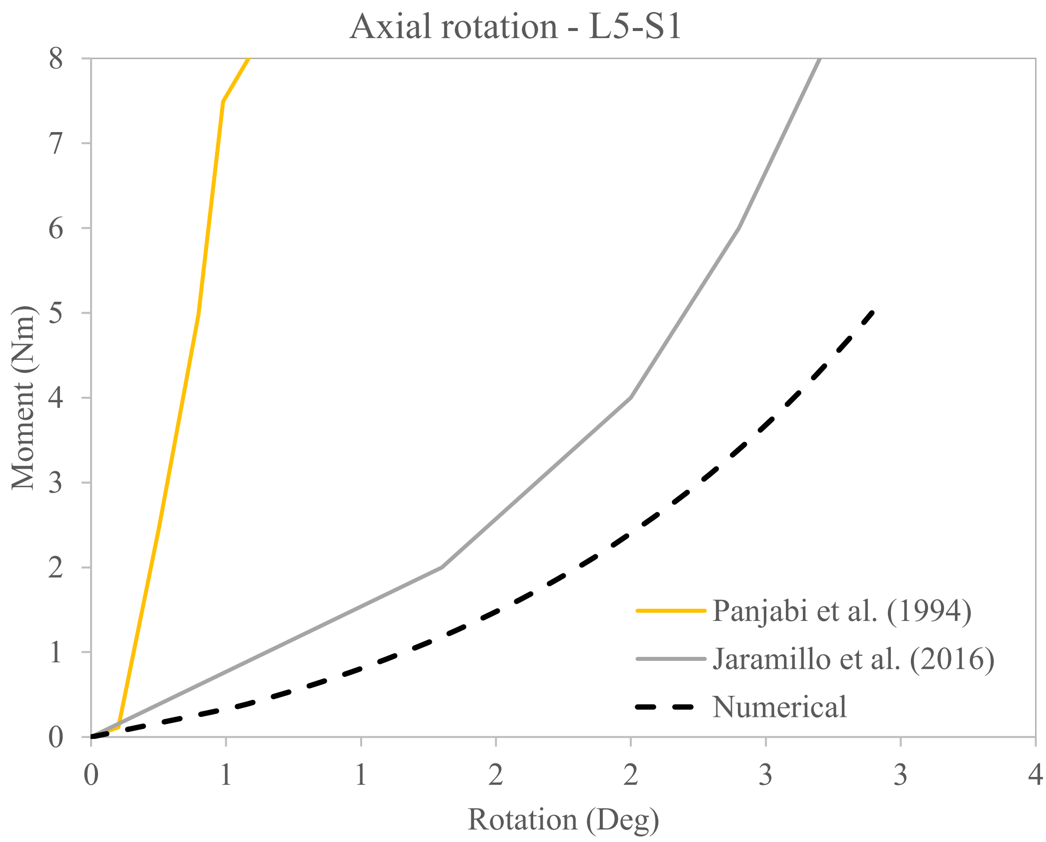
Axial rotation - L5-S1
Moment (Nm)
Rotation (Deg)
0
1
2
3
4
5
6
7
8
0
1
1
2
2
3
3
4
Panjabi et al. (1994)
Jaramillo et al. (2016)
Numerical

References

[1] L.F. Nicolini, J. Greven, P. Kobbe, F. Hildebrand, M. Stoffel, B. Markert, B.M. Yllera, M.S. Simoes, C.R. de M. Roesler, E.A. Fancello, The effects of tether pretension within vertebral body tethering on the biomechanics of the spine: a Finite Element analysis, Latin American Journal of Solids and Structures 19 (2022). https://doi.org/10.1590/1679-78256932.

[2] L.F. Nicolini, The Effects of Vertebral Body Tethering System on the Biomechanics of the Thoracolumbar Spine, Federal University of Santa Catarina and RWTH Aachen University, 2023. https://doi.org/10.18154/RWTH-2023-04766.

[3] M. Couvertier, A. Germaneau, M. Saget, J.-C. Dupré, P. Doumalin, F. Brémand, F. Hesser, C. Brèque, M. Roulaud, O. Monlezun, T. Vendeuvre, P. Rigoard, Biomechanical analysis of the thoracolumbar spine under physiological loadings: Experimental motion data corridors for validation of finite element models, Proc Inst Mech Eng H 231 (2017) 975–981. https://doi.org/10.1177/0954411917719740.

[4] A. Germaneau, T. Vendeuvre, M. Saget, P. Doumalin, J.C. Dupré, F. Brémand, F. Hesser, M. Couvertier, C. Brèque, P. Maxy, M. Roulaud, O. Monlezun, P. Rigoard, A novel approach for biomechanical spine analysis: Mechanical response of vertebral bone augmentation by kyphoplasty to stabilise thoracolumbar burst fractures, J Mech Behav Biomed Mater 59 (2016) 291–303. https://doi.org/10.1016/j.jmbbm.2016.02.002.

[5] Y. Guan, N. Yoganandan, J. Moore, F.A. Pintar, J. Zhang, D.J. Maiman, P. Laud, Moment-rotation responses of the human lumbosacral sinal column, J Biomech 40 (2007) 1975–1980. https://doi.org/10.1016/j.jbiomech.2006.09.027.

[6] F. Heuer, H. Schmidt, Z. Klezl, L. Claes, H.-J. Wilke, Stepwise reduction of functional spinal structures increase range of motion and change lordosis angle, J Biomech 40 (2007) 271–280. https://doi.org/10.1016/j.jbiomech.2006.01.007.

[7] H.E. Jaramillo, C.M. Puttlitz, K. McGilvray, J.J. García, Characterization of the L4–L5–S1 motion segment using the stepwise reduction method, J Biomech 49 (2016) 1248–1254. https://doi.org/10.1016/j.jbiomech.2016.02.050.

[8] H.-J. Wilke, S. Grundler, C. Ottardi, C.-E. Mathew, B. Schlager, C. Liebsch, In vitro analysis of thoracic spinal motion segment flexibility during stepwise reduction of all functional structures, European Spine Journal 29 (2020) 179–185. https://doi.org/10.1007/s00586-019-06196-7.

[9] H.-J. Wilke, A. Herkommer, K. Werner, C. Liebsch, In vitro analysis of the segmental flexibility of the thoracic spine, PLoS One 12 (2017) e0177823. https://doi.org/10.1371/journal.pone.0177823.

[10] A. Beckmann, Biomechanical Investigation of Posterior Dynamic Stabilization Systems of the Lumbar Spine, RWTH Aachen University, 2021. https://doi.org/10.18154/RWTH-2021-05091.

[11] A. Beckmann, C. Herren, L.F. Nicolini, D. Grevenstein, S. Oikonomidis, P. Kobbe, F. Hildebrand, M. Stoffel, B. Markert, J. Siewe, Biomechanical testing of a polycarbonate-urethane-based dynamic instrumentation system under physiological conditions, Clinical Biomechanics 61 (2019) 112–119. https://doi.org/10.1016/j.clinbiomech.2018.12.003.

[12] A. Beckmann, L.F. Nicolini, D. Grevenstein, H. Backes, S. Oikonomidis, R. Sobottke, P. Kobbe, F. Hildebrand, M. Stoffel, B. Markert, J. Siewe, C. Herren, Biomechanical in vitro test of a novel dynamic spinal stabilization system incorporating polycarbonate urethane material under physiological conditions, J Biomech Eng 142 (2020). https://doi.org/10.1115/1.4044242.
